# Proof of Concept for a Controlled Raman-Compatible Skin-Mimicking Hydrogel Substrate for Chemical Imaging Technique Development

**DOI:** 10.3390/molecules31091530

**Published:** 2026-05-05

**Authors:** Kevser Kemik, Charlotte De Bleye, Pierre-Yves Sacré, Philippe Hubert, Eric Ziemons

**Affiliations:** 1 Laboratory of Pharmaceutical Analytical Chemistry, ViBra-Santé HUB, CIRM, Department of Pharmacy, University of Liege (ULiege), Avenue Hippocrate 15, 4000 Liège, Belgium; cdebleye@uliege.be (C.D.B.); ph.hubert@uliege.be (P.H.); 2 Research Support Unit in Chemometrics, CIRM, Department of Pharmacy, University of Liege (ULiege), Avenue Hippocrate, 15, 4000 Liège, Belgium; pysacre@uliege.be

**Keywords:** chemical imaging, Raman imaging, SERS chemical imaging (SER-CI), hydrogel substrate, model characterization, mixed modelling, drying kinetics, spatial homogeneity, silver nanoparticles (AgNPs)

## Abstract

The quality of Surface-Enhanced Raman Chemical Imaging (SER-CI) rely on several parameters, among which the uniform deposition of metallic nanoparticles impacts greatly the result. Optimizing deposition protocols for biological samples is challenging due to inherent spatial heterogeneity, preventing the distinction between deposition artefacts and true analyte distribution. However, to optimize the deposition parameters, it is necessary to have a controlled experimental model. This study presents the development of a repeatable dried gelatine–agarose hydrogel as a controlled analytical substrate with the uniform spatial homogeneity of diphenhydramine hydrochloride as the experimental model for further nanoparticle deposition optimization. With its skin-mimicking Raman fingerprint, the proposed hydrogel enables the systematic evaluation of deposition techniques without biological variability. Confocal Raman imaging performances are as follows: the normalization-based ratio (I_1003_/I_1469_) achieved an intra-day RSD of 3.6–8.2%, inter-day RSD of 6.5%, and intra-day pixel-wise RSD (%) of 8.3–12.3%. The Distribution Homogeneity Index (DHI) confirmed the analyte’s uniform distribution. Drying kinetics modelling revealed a diffusion-based dehydration process, with repeatable batch production. Application of dried hydrogels for SERS chemical imaging confirmed diphenhydramine hydrochloride detectability inside the polymeric matrix, with the proportionality of intensity based on the diphenhydramine hydrochloride concentration. A preliminary performance comparison of nanoparticle deposition by drop-casting and spray-coating demonstrates the applicability of the developed model. This standardized matrix provides a reference platform for evaluating deposition homogeneity, distinguishing method performance from sample artefacts and accelerating chemical imaging method development and performance through optimization.

## 1. Introduction

Pharmaceutical research on topical drug development represents one of the fundamental steps in translating drug formulations from laboratory concepts to real clinical applications [1]. Among them, one of the research and development steps in this particular area that stands out is the in vitro permeation testing (IVPT) [2], crucial for the first evaluation of developed formulations. Current regulatory institutions such as the Organisation for Economic Co-operation and Development (OECD) and the European Medicines Agency (EMA) have established comprehensive guidelines for implementing diffusion mimicking protocols, in which the Franz diffusion cells are used to simulate real-life conditions in topical formulation applications [3,4,5]. These standardized methods enable the diffusion of active compounds through various skin models, including real human skin tissues obtained majorly from surgical donations, porcine skin for its similarity to real human skin, or other comparable biological models [2].

Practically, the recollected receptor and donor fluids from these permeation studies are subjected to analysis, primarily based on separative techniques such as High-Performance Liquid Chromatography (HPLC) to quantify total permeated drug concentrations [6,7]. While this approach represents the current gold standard as preconized by the OECD and EMA guidelines for its ease of implementation, it provides however no spatial information about drug distribution and no temporal behaviour during the permeation process [8]. Advanced analytical methods are increasingly being explored to overcome these limitations, with trends in analytical chemistry pointing towards more sophisticated approaches [9]. Still, challenges remain as other current methods face problems, including the inability to provide spatial distribution information and limited sensitivity for low-dose formulations [10,11]. There is therefore an urgent need for advanced chemical imaging techniques that can acquire missing experimental information, therefore bringing the spatial data as complementary information for the evaluation of drug formulations [1].

Several alternative imaging techniques have been developed for pharmaceutical analysis including fluorescence-based microscopy [12], Raman imaging [13,14,15,16,17], and Matrix-Assisted Laser Desorption Ionization Mass Spectroscopy Imaging (MALDI-MSI) [18,19]. On the one hand, fluorescence microscopy, especially coveted in cell metabolomics, offers several advantages including high sensitivity and selectivity through the use of dyes, spatial resolution, and real-time imaging capabilities [12], but it presents inherent limitations due to its dependence on fluorescent dyes that may influence drug properties (among which is permeability), thus possibly introducing artefacts into permeation studies. Raman imaging (R-CI) has emerged as a promising label-free imaging technique. Indeed, Raman imaging does not necessitate analyte labelling via chemical derivatization, eliminating potential interference in drug permeation studies [14,15], and it is non-destructive and provides detailed structural information about drug distribution within biological matrices [13,20,21,22]. However, conventional Raman techniques face significant sensitivity limitations, particularly when analyzing low-dose formulations used in topical drug applications [7,20,21,22,23,24] requiring long acquisition times. Similarly, MALDI-MSI offers the advantage of label-free detection with limited spatial resolution for drug distribution mapping in skin tissues, enabling simultaneous quantitative and qualitative analyses of multiple compounds, but faces limitations including tissue matrix effects, complex sample preparation requirements, and potential analyte suppression effects that can affect quantification accuracy [18,25,26].

On the other hand, Surface-Enhanced Raman Chemical Imaging (SER-CI), an alternative analytical technique derived from R-CI, is increasingly implemented to circumvent the described major limitations [27,28,29,30], being interferences from fluorescence and weak sensitivity [31,32]. However, SER-CI requires deposition of a metallic nanoparticle (NP) substrate to enhance the Raman signal, making the sample preparation step crucial for the application of this approach [33,34]. Currently, SER-CI implementation faces specific obstacles coming from sample variability, including the surface inhomogeneity of the molecule of interest, leading to the cracking of the sample [35,36,37,38] and/or the coffee-ring effect [39,40], variations in sample thickness, and concentration gradients, all of which can distort the spatial distribution image since the SERS signal is dependent of sample quality, as well as NP concentration and covering quality [41,42]. Recent developments in SERS substrates and nanoparticle synthesis, including optimized NP synthesis by several approaches [43,44,45,46], have opened new possibilities for diverse applications, by reducing the variability induced by NP batches [44,46,47]. Additionally, the covering must be consistently homogeneous in distribution across the sample surface to enable the localized enhancement of the Raman signal [41,48]. These sample preparation steps are often prone to variability, introducing a major source of uncertainty in both signal intensity and spatial accuracy [29,47]. Current sample preparation protocols include drop-casting [49,50,51], absorption coating [41], spin-coating [52], laser-induced depositing [53], and spray-coating techniques [41,48,54,55] to improve SER-CI performance [42,56,57], all of which require dedicated optimization before undertaking the analysis of real samples. This need extends beyond skin studies as any soft matrix system subjected to SER-CI analysis would benefit from such a controlled and reproducible reference standard for deposition method development and optimization. In this context, a critical yet often overlooked step is the development of a well-designed, well-characterized, and repeatable model matrix that can serve as a universal standard for these deposition approaches, to optimize the SER-CI sample preparation step, while also being applicable in the context of biomedical studies.

The need to optimize sample preparation processes suitable for dermatological studies has led to the development of matrices that can mimic skin complexity [58,59,60,61]. A central and distinctive feature of the current polymeric matrix lies in its spectral behaviour: Raman bands of carefully designed polymeric matrices can closely reproduce those most frequently encountered in biological tissues, particularly the skin, including characteristic bands associated with proteins, lipids, and structural biomolecules [2]. This spectral resemblance provides a realistic and representative chemical fingerprint without the inherent complexity and variability of native tissue, making such matrices reliable and tuneable standards for early-stage method development. Beyond spectroscopic relevance, these matrices offer structural simplicity, repeatability, and compatibility with nanoparticle deposition protocols, enabling the systematic evaluation of signal homogeneity, spatial resolution, and SERS substrate coverage in fully controlled settings. This workflow could ultimately enable the tracking of low-dose active pharmaceutical ingredients or biomarkers within biological matrices and could be extended to other imaging techniques requiring homogeneous reference standards.

In this context, the present study aimed at developing a controlled dried polymeric matrix with Raman spectral skin-mimicking properties, loaded with a model API that could serve as a reliable reference standard for advanced spectroscopic technique development for topical drug studies [62,63,64]. To support the development of a robust analytical platform, Critical Quality Attributes (CQAs) were defined to frame the expectations and guide the evaluation of the matrix, focusing on spatial consistency, preparation repeatability, and compatibility with chemical imaging acquisition techniques. The developed matrix was systematically evaluated for spatial homogeneity, spectral skin mimicry capabilities, and preparation repeatability using both analytical metrics and statistical approaches.

## 2. Results and Discussion

### 2.1. Matrix Design

Polymers used to design the model matrix present several advantages, which have been described previously. However, according to their physicochemical properties, the compatibility with APIs is not universal and needs to be studied. To focus specifically on matrix–API compatibility, APIs were selected using four pragmatic physicochemical descriptors: formulation pH after API addition, aqueous solubility, molecular weight and lipophilicity described with the logarithm of the partition coefficient P (logP). These descriptors were used to anticipate API behaviour during dehydration through effects on API–matrix interactions and drying-driven redistribution, while keeping the preparation and drying conditions unchanged.

Candidate APIs were pre-screened to remain fully dissolved at the working concentration, as precipitation and crystallization during solvent evaporation can generate spatial artefacts and bias apparent heterogeneity. Evaporation-driven transport phenomena are well documented to cause non-uniform deposition patterns when solutes redistribute during drying [21]. In parallel, the hydrogel pH after API incorporation was targeted to remain in a moderately acidic range because gelatine gel properties are pH-dependent and can weaken outside an appropriate window of pH, i.e., between 5 and 8 [36]. Additionally, if used in its salt form, the selected API can ensure sufficient aqueous solubility, especially during the drying process of the hydrogel, thereby preserving gel integrity by preventing crack formation and crystallization.

Molecular weight and logP were therefore treated as screening variables rather than sole predictors of mobility, since solute transport in hydrogels is governed by network mesh size and specific drug–polymer interactions, highly dependent on the system ionic strength [65]. Notably, the agarose component is primarily hydrophilic, so hydrophobic binding is mainly attributed to gelatine domains and to interaction chemistry rather than to the agar itself [66].

Based on these criteria, diphenhydramine hydrochloride was found to generate adequate Raman response and structural stability. Diphenhydramine hydrochloride is a weak base, in its salt form, with a pKa around 9 [67]; thus, it is expected to be predominantly protonated under the mildly acidic formulation conditions [36]. Consequently, a strong electrostatic attraction between gelatine and the protonated forms of the selected APIs is not anticipated [68]. These observations also indicate that matrix–API compatibility requires a multi-descriptor evaluation rather than reliance on any single physicochemical predictor. Gel integrity was preserved upon diphenhydramine hydrochloride incorporation, with no visible liquefaction, cracking, or crystallization under the selected preparation conditions. In parallel, the formulation pH after API addition remained in the mildly acidic range with a value of 5.27, compatible with the gel structure.

**Figure 1:**
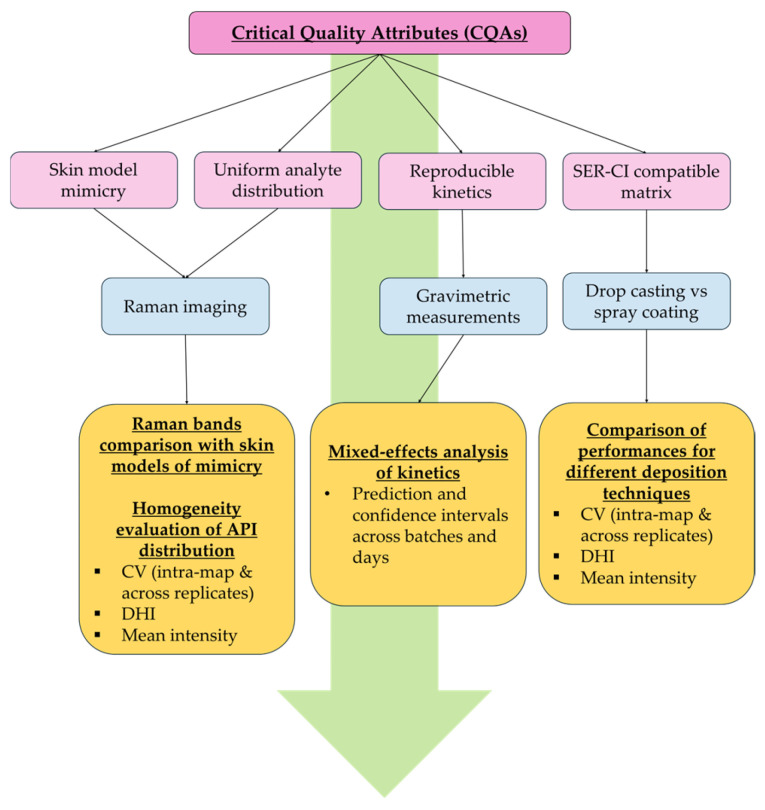
Design framework for the development of a dried agarose–gelatine hydrogel standard for Surface-Enhanced Raman Chemical Imaging (SER-CI). Four Critical Quality Attributes (CQAs) were defined: (i) skin mimicry assessed by Raman imaging and evaluated through band position comparison; (ii) uniform analyte distribution evaluation, assessed by Raman imaging and evaluated through relative standard deviation (RSD%) and the Distribution Homogeneity Index (DHI), and mean intensity across intra-map and inter-replicate measurements; (iii) reproducible drying kinetics and homogeneous dehydration, monitored by gravimetric measurements to predict drying behaviour and associated confidence intervals across batches and days; and (iv) SER-CI matrix compatibility, assessed by comparing drop-casting and spray-coating deposition techniques.

Experiments were performed over three independent days, with nine hydrogels prepared per day, yielding a total of 27 replicates (*n* = 27) per batch. Dried hydrogel performance was assessed based on the CQAs using (i) Raman imaging for band comparison between dried hydrogels and reconstructed skin model Episkin^®^ RHE for the skin mimicry evaluation; (ii) Raman imaging using ratiometric band normalization $I_{1003{cm}^{-1}}/I_{1469{cm}^{-1}}$ to mitigate thickness- and focus-related intensity variability issues, combined with the spatial homogeneity assessment of API distribution; (iii) gravimetric drying curves analyzed to evaluate repeatability and to characterize drying kinetics and finally; and (iv) preliminary compatibility testing for SER-CI method development using the polymeric matrix as a standard, to pre-evaluate the analytical performance of using these types of standards as a proof of concept. This is presented in Figure 1.

### 2.2. Spectral Evaluation of Dried Hydrogels

#### 2.2.1. Skin Mimicry of Hydrogels

**Figure 2:**
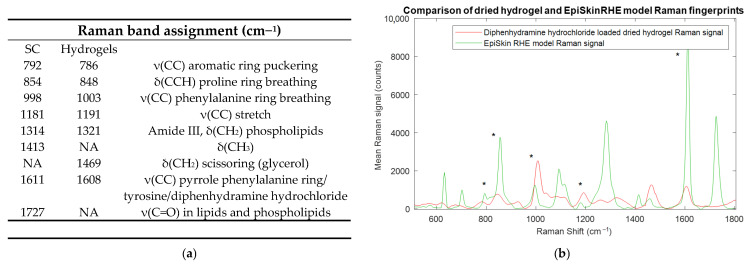
Raman spectra and band assignments of the polymeric matrix compared to EpiSkin^®^ stratum corneum. (**a**) Band correspondence table between EpiSkin^®^ stratum corneum (SC) and the agarose–gelatine hydrogel matrix; N/A indicates bands absent from the matrix spectrum. ν, stretch; δ, deformation; ρ, rock. (**b**) Representative Raman spectra of the solid polymeric matrix containing diphenhydramine hydrochloride (red) and EpiSkin^®^ control (green) recorded in the 500–1800 cm^−1^ region. * Highlighted bands indicate spectral features common to both the EpiSkin^®^ RHE SC and the dried model matrix.

One of the primary objectives of this work was to develop a dried hydrogel standard capable of generating a Raman fingerprint signal similar to those encountered in biomedical matrices, especially human skin tissue. The EpiSkin^®^ RHE model, a commercially available 3D human epidermis model, was used as the spectral reference to guide matrix design and evaluate spectral concordance [2,17]. The design framework is summarized in Figure 2. The Raman band assignment table shows that the solid polymeric matrix reproduced some of the principal spectral features of EpiSkin^®^ RHE stratum corneum (SC) across the biologically relevant fingerprint region, from 500 to 1800 cm^−1^, supporting its suitability as a skin-mimicking standard for Raman imaging and derived techniques [2,22]. This specific combination was selected to ensure complementary functional requirements: agar contributes to a rigid polysaccharide network that confers mechanical stability to the dried film and governs gel-setting behaviour, providing a structurally robust scaffold that resists crack formation upon drying [69]. Gelatine, as a hydrolysed collagen derivative rich in polypeptide sequences, is the primary driver of the skin-mimicking Raman fingerprint, given its protein-based composition and well-documented spectral analogy with structural skin proteins, including collagen and keratin [70,71]. Glycerol, on the other hand, serves as a plasticizer: by intercalating within the polymer network and disrupting intermolecular hydrogen bonding between polymer chains, it lowers the glass transition temperature of the dried hydrofilm and dissipates the mechanical stress that develops during water evaporation, preventing cracks [72]. Formulations prepared without glycerol exhibited surface cracking upon evaporation equilibrium, whereas glycerol-containing films dried while maintaining macroscopic integrity across replicates, confirming experimentally the plasticising effect of glycerol in the matrix.

**Figure 3:**
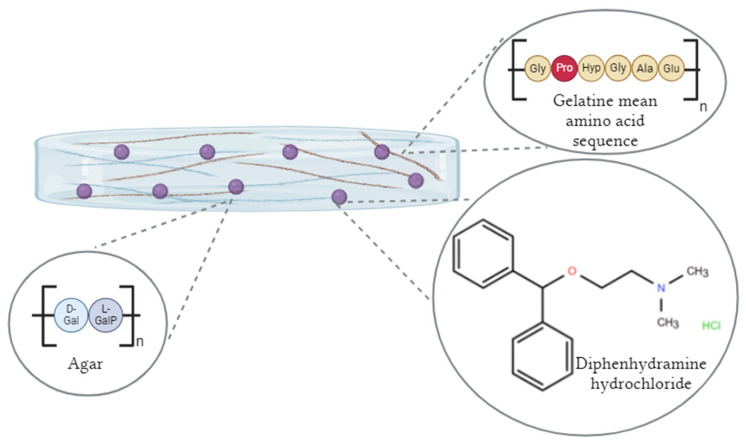
Schematic representation of the hydrogel containing agar, gelatine and diphenhydramine hydrochloride. The molecular structure of diphenhydramine hydrochloride was drawn using the Ketcher online chemical structure editor (EPAM Systems) and the main amino acid sequence of gelatine and the monomer of agar using Canva^®^. Gly: glycine; Pro: proline; Hyp: hydroxyproline; Ala: alanine; Glu: glutamic acid for gelatine. D-Gal: D-galactose; L-GalP: 3,6-anhydro-L-galactopyranose for agar.

The spectral similarity between the matrix and EpiSkin^®^ RHE SC is grounded in their shared protein-based composition. EpiSkin^®^ RHE SC is primarily constituted of normal human keratinocytes organized in stratified layers rich in structural proteins such as keratins, filaggrin and lipid–protein assemblies, while the hydrogel matrix presents polypeptide backbone signatures derived from its gelatine component, with agar contributing to the structural support. The compositional analogy between gelatine and the EpiSkin^®^ model underlies the observed band correspondence. The band near 792 cm^−1^ in the SC of EpiSkin^®^ RHE, assigned to aromatic ring puckering, is observed at 786 cm^−1^ in the matrix at a comparatively lower intensity, consistent with differences in the relative abundance of aromatic residues between the two matrices [22,73]. Proline-associated band highlighted in red, as seen in Figure 3 and represented at 854 cm^−1^ in the EpiSkin^®^ RHE SC spectra, is reproduced at 848 cm^−1^ in the matrix, reflecting the presence of proline-containing peptide sequences in gelatine and in the skin model [70]. Amide III and CH_2_ phospholipid contributions, at 1314 cm^−1^ in the SC of EpiSkin^®^ RHE, appear at 1321 cm^−1^ in the matrix, in keeping with the amide backbone common to collagen-derived proteins [22,70,71,73]. Phenylalanine ring/tyrosine stretch (1611/1608 cm^−1^) bands, present in low proportions in gelatine, are still similarly reproduced in comparison with the Episkin^®^ RHE SC with minor wavenumber shifts attributed to differences in the secondary structure and the local chemical environment [22,73]. This assignment could also be linked to diphenhydramine hydrochloride, as its structure contains two phenyl rings, presenting a characteristic band in this spectral region, as represented in Figure 3.

#### 2.2.2. Raman Detectability

Spectral selectivity was established by comparing mean R-CI maps from loaded hydrogels to (i) the raw compound in the powder form and (ii) a blank hydrogel prepared with gelatine–agarose and glycerol without API.

**Figure 4:**
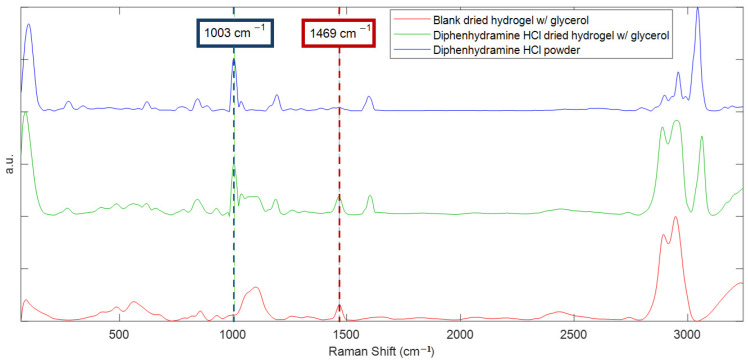
Tracer band selection and internal reference for diphenhydramine HCl in gelatine–agarose hydrogels. Raman spectra of a blank hydrogel with glycerol (red), diphenhydramine HCl-loaded hydrogel (green), and the raw diphenhydramine HCl powder (blue). The phenyl ring breathing near 1003 cm^−1^ (dashed blue line) is prominent in the powder and retained in the loaded hydrogel, while absent in the blank, confirming its use as the analyte tracer band. The 1469 cm^−1^ band (dashed red line) is matrix-dominated by glycerol and appears consistently in both the blank and loaded hydrogel; it is used as the matrix-assigned tracer (internal reference) for $I_{ratio}$.

The diphenhydramine hydrochloride-loaded hydrogel exhibits a distinct band at 1003 cm^−1^, assigned to the phenyl ring breathing mode, also observed in the raw powder, and absent in the blank condition [6,74], as illustrated on Figure 4. This three-way comparison satisfies a priori requirements for a tracer band, attesting matrix selectivity with no false positive in the blank condition, and the chemical assignment, meaning that the raw powder tracer band and hydrogel tracer band match, attesting the assignment of the band to the API. Because the 1003 cm^−1^ band is intense and isolated in the spectral range, and because the blank lacks a co-located feature, 1003 cm^−1^ band was selected as the analyte tracer band for the hydrogel.

To improve the comparability across gels during Raman mapping, diphenhydramine hydrochloride signals were exploited both as raw band intensities and as the normalized ratio to correct minor thickness and focus variations. Normalization used a matrix-assigned tracer band that satisfies the selectivity requirement, meaning that no false positives should be observed in the raw diphenhydramine powder but should be present in both the blank condition and API-loaded hydrogel. Therefore, the 1469 cm^−1^ band, assigned to glycerol, met these requirements and showed good spectral resolution. Therefore, the $I_{1003{cm}^{-1}}/I_{1469{cm}^{-1}}$ ratio was retained for the subsequent hydrogel evaluation and was referred to as $I_{ratio}$.

The diphenhydramine hydrochloride tracer band was then subjected to sensitivity analysis by computing the signal-to-noise (S/N) ratio of the 1003 cm^−1^ phenyl ring breathing band on the baseline-corrected spectra. The pixel-wise obtained median S/N was 221, indicating robust signal quality suitable for reliable ratiometric imaging across the hydrogel maps. This criterion was crucial in the framework of this study as it suppresses the risk of evaluation bias due to low S/N ratio values for the homogeneity assessment of the model matrix, compromising our interpretation. The details on the S/N ratio diagnostics are provided in the Supplementary Data (Supplementary Note S1; Figure S1).

### 2.3. Spatial Homogeneity

The successful application of intensity normalization relies on two critical assumptions: (i) the uniform co-incorporation of API and glycerol tracer during hydrogel formation, and (ii) proportional signal attenuation for both species under defocusing conditions. Three metrics were extracted from normalized maps: (i) mean normalized intensity ($I_{ratio}$), (ii) relative standard deviation (RSD%), and (iii) Distribution Homogeneity Index (DHI). These metrics were calculated on identical pixel sets for both development and validation datasets. All used data are available in Supplementary Data (Supplementary Note S2; Tables S1–S4).

Variance metrics for the development set (27 hydrogels across three batches over 3 days, *p* = 9 gels/day) are summarized in Table 1. As displayed, $I_{ratio}$ ranged from 2.62 to 2.67 across all study days, with intra-day RSDs of 3.55–8.15% and inter-day RSD of 6.47%. The consistent $I_{ratio}$ across replicates and days demonstrates reproducible tracer incorporation and stable matrix composition.

Intra-day spatial distribution RSD (%) ranged from 8.30 to 12.33, while inter-day RSD (%) was 10.53. Spatial homogeneity was evaluated using the Distribution Homogeneity Index (DHI) metric calculated via the CLMB procedure [75]. The DHI compares the variance profile of experimental intensity distribution across multiple spatial scales to that of randomized simulated maps; a DHI close to 1 indicates perfect spatial homogeneity, while a DHI above 1 reflects increasing clustering, translating non-homogeneous API distribution. The observed mean DHI values per day ranged from 1.14 to 1.24, close to the ideal value of 1, confirming nearly uniform API distribution.

To confirm the robustness of the normalization approach, an independent confirmation batch (*n* = 3 gels) was prepared and analyzed under identical conditions as illustrated in Table 2. Intensity precision RSD was 1.41%, pixel-wise RSD was 9.74%, and mean DHI for the batch was 1.16, similar to the previously observed metric performance. A low observed intra-batch RSD demonstrates preparation reproducibility.

**Figure 5:**
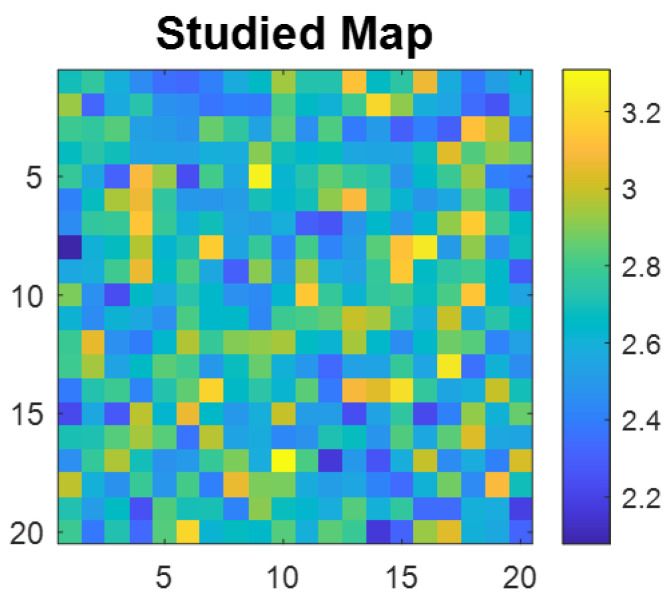
Homogeneity assessment of the dried hydrogel surface by R-CI. Studied map of pixel intensities across the hydrogel surface after normalization. The colour scale represents normalized Raman intensity, where blue pixels indicate lower intensity values and yellow pixels indicate higher intensity values. The even spatial distribution of pixel intensities across the map attests to the homogeneity of the hydrogel surface.

The DHI values, as observed for one of the confirmatory batches in Figure 5, confirm that diphenhydramine hydrochloride present in the dried hydrogels does not diffuse or crystallize during gelation or drying, consistent with molecular-level dispersion in the polymer matrix. This is further supported by the DHI value obtained for one of the three confirmatory batch sample with a value of 1.12. This represents a prerequisite for reproducible nanoparticle deposition or matrix application in subsequent SERS imaging experiments. The observed intra-day variability likely reflects minor differences in gel thickness or surface topography, while inter-day variability incorporates additional sources such as preparation difference, ambient humidity affecting drying kinetics or slight instrumental drift. Nevertheless, all sources of variation remain well controlled within pharmaceutical analytical specifications, validating the hydrogel platform as a fit-for-purpose calibration standard for imaging method development.

### 2.4. Repeatability of Drying Kinetics

The experimental equilibrium time was determined as the point at which the absolute change in relative water content (RWC) between consecutive hourly measurements was ≤0.1%, sustained over multiple points and determined experimentally. Above this criterion, the variation in RWC for the following hours did not reach a stabilization plateau. Applying this criterion, the dehydration plateau was reached after 20 h based on the RWC for all replicates. During the final three hours following equilibrium, the across-replicate variability, all day combined, was minimal with the RSD% oscillating between 0.04% and 0.60%, confirming a stable end-state suitable for subsequent imaging. Drying kinetics profiles across batches show similar, concave-down drying curves that converge to a common endpoint of RWC at 22.29% ± 0.43, under the fixed drying conditions of 25 °C/60% RH. The shape of the curves is very characteristic of a transition from a brief surface-controlled phase to a diffusion-controlled phase [76]. This equilibrium is highly dependent on water removal mechanisms that are governed by the vapour pressure gradient between the hydrogel and the surrounding air, and the controlled condition in the incubator maintains a consistent external driving force and prevents local humidity build-up [38]. As dehydration proceeds, the network consolidation of the gelatine–agarose matrix increases transport resistance with the formation of a thin, dense surface layer, decreasing the effective diffusivity of water. This results in the dropping of the evaporation rate as the equilibrium is approached [38]. The non-zero equilibrium RWC reflects the moisture sorption of the matrix, depending on the external conditions and the composition of the matrix, mainly due to the humectant characteristic of glycerol and hygroscopic characteristic of gelatine [77,78]. The reproducible end-state provided a consistent baseline for the subsequent analytical evaluations.

Hierarchical structure of the drying kinetics dataset was solved by mixed-effects modelling, where repeated measures were obtained over time from multiple gels in different batches, across different days. The homogeneous antedependence covariance (ANTE-EV) structure was used for the drying kinetics evaluation.

The ANTE-EV structure is adequate in modelling decaying correlations across time intervals, where observations are most strongly correlated with their immediate predecessors, and this correlation decreases with the increasing temporal distance. This structure is especially accurate for relatively small datasets such as the present one, where models with many parameters may not converge or yield unstable estimates [79].

To be able to construct a linear model with a polynomial-like decaying dataset, relative water content (RWC) was log-transformed to stabilize variance and obtain a normalized distribution of values in the datasets. Model adequacy was assessed through residual diagnostics and normality checks. Examination of the conditional residuals relative to predicted log(RWC) values for the normality and homoscedasticity assessment reveals no systematic deviation from the reference line and suggests that the model residuals are symmetrically distributed around zero without any evidence of a visible structure. Model data and model adequacy diagnostics are provided in detail in the Supplementary Data (Supplementary Notes S3 and S5; Tables S5–S7 and S9).

Interpretation of fixed effects, in Table 3, assessed through F-test on the datasets, permitted the extraction of the following results. Firstly, the intercept, corresponding to the plateau of equilibrium observed, is −1.5066 ± 0.0136, reflecting the fact that RWC was modelled on a log scale, and converting to RWC in % gives a value of $e^{-1.51}$, i.e., 22.29% ± 0.30, and it proves the presence of a repeatable plateau of RWC across the batches and the replicates. Additionally, the comparison of time points with the last time point confirms the equilibrium plateau: all time contrasts versus the last time are highly significant with a *p*-value of <0.001 (cut-off placed at 0.05), with a non-significance between the last two time points (*p* = 0.42 and 0.47), rejecting the H_0_ hypothesis that these time points are significantly different from each other.

Concerning the day-induced variability, considering day 1 as reference batch, no significant inter-day difference was observed between day 1 and day 2 (*p* = 0.60), day 1 and day 3 (*p* = 0.64), and day 2 and day 3 (*p* = 0.95), all significantly superior to the cut-off of 0.05. One point of attention is also the standard error in the lower range compared to the estimate factor of the model, proving that the datasets do not highly diverge across samples.

Residual diagnostics are presented and described in the Supplementary Data (Supplementary Note S6; Figure S4). Another approach of modelling was also considered following the bimodal Weibull design, also described in the Supplementary Data (Supplementary Note S4; Equation (S1), Figures S2 and S3 and Table S8), which includes the model equation and methodology [80,81,82,83,84,85,86].

### 2.5. Compatibility of the Matrix with SERS Imaging

**Figure 6:**
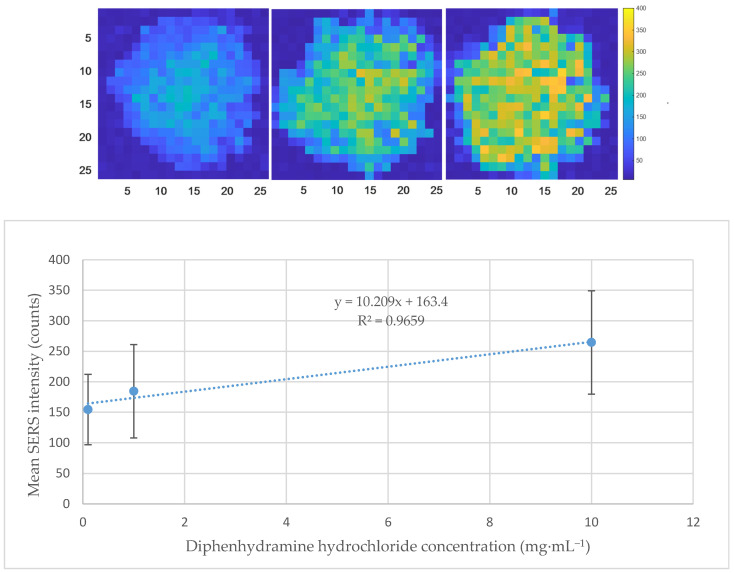
Concentration-dependent mean SERS intensity response of dried diphenhydramine hydrochloride hydrogel (*n* = 3 per concentration level) and their respective generated SER-CI map, from left to right, 0.1, 1 and 10 mg·mL^−1^ (same intensity scale for comparison). In the SER-CI maps, blue and yellow pixels represent low and high SERS intensity values, respectively. The progressive shift towards higher intensity values with the increasing concentration is consistent with the concentration-dependent SERS response.

Compatibility of the dried diphenhydramine hydrochloride hydrogel matrix with SERS imaging was assessed by evaluating the mean SERS intensity response across three concentration levels (0.1, 1 and 10 mg·mL^−1^) using a silver nanoparticle (AgNP) suspension, synthetized according to the optimized microwave-assisted protocol from Horne et al. [44]. Nanoparticles were concentrated by centrifugation and by removing the respective supernatant to obtain 10-times relatively concentrated AgNPs, which were then deposited by drop-casting, with three replicates per concentration level. As observed in Figure 6, a linear relationship was established between the diphenhydramine hydrochloride concentration and mean SERS intensity, described by the equation y = 10.209x + 163.4 with a coefficient of determination R^2^ = 0.9659, indicating a proportional API concentration-dependent SERS response across the tested range.

This result confirms that the hydrogel matrix does not suppress or interfere with the SERS signal generated by the AgNPs, supporting its compatibility for SER-CI applications. The positive slope of the regression further demonstrates that an increasing analyte concentration is detectable within the matrix. Nevertheless, the high standard deviations observed across replicates, most notably at 10 mg·mL^−1^, are attributed to the inherent variability of the drop-casting deposition technique. As illustrated in Figure 6, SERS intensity maps based on the diphenhydramine hydrochloride band at 1003 cm^−1^ of the three replicates from 0.1 to 10 mg·mL^−1^ reveal a characteristic spatial distribution pattern, with higher signal intensity concentrated in the centre of the droplet and a progressive decrease towards the edges, commonly associated with drop-casting, as well as intensity proportional to the respective concentration of diphenhydramine hydrochloride. This non-uniform AgNP distribution across the hydrogel surface directly translates into high intra- and inter-replicate variability, highlighting the limitation of drop-casting as a deposition method and supporting the transition towards a more controlled and reproducible AgNP application approach, such as spray-coating, in subsequent experimental phases. Used data can be found in Supplementary Data (Supplementary Note S7; Table S10).

**Figure 7:**
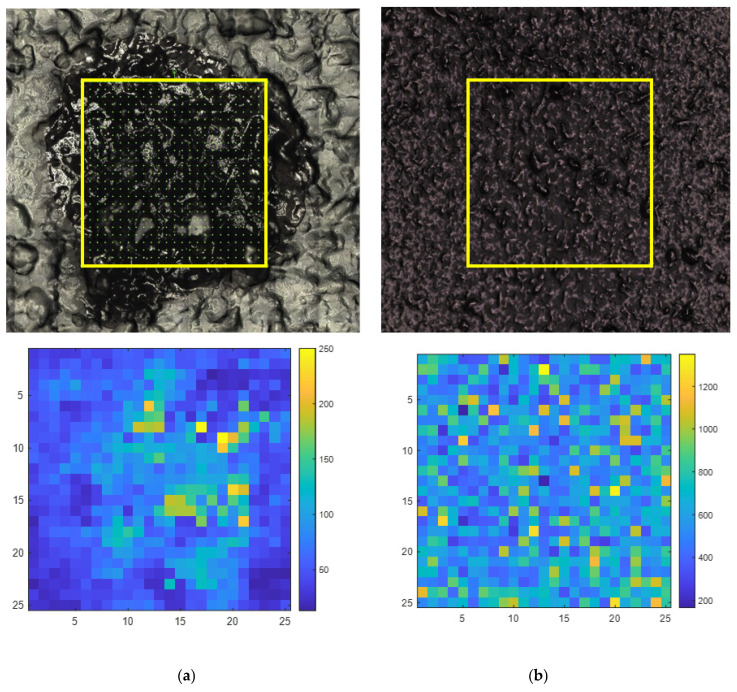
Direct homogeneity comparison between drop-casting (**a**) and spray-coating (**b**) of AgNPs on dried diphenhydramine hydrochloride hydrogel surface. For each condition: (**Top**) mapping area image covered sample, highlighted by the yellow box. (**Bottom**) Studied map of pixel intensities. The colour scale represents Raman intensity, where blue pixels indicate lower intensity values and yellow pixels indicate higher intensity values. The spatial distribution of pixel intensities across the map allows for a direct visual comparison of the homogeneity achieved between the two AgNP deposition methods.

A comparison with spray-coating deposition, as presented in Figure 7, further contextualizes the limitations of drop-casting through a direct comparison. First, on the image of the covering area, a mapping zone equivalent to a 25 × 25-pixel square, highlighted in bright yellow, was selected to ensure fair comparison, with it only containing well-covered areas. The drop-cast sample yielded a mean DHI value of 3.03, a mean SER-CI intensity of 68.1 with a standard deviation of 38.7 and an RSD% of 56.81%. In contrast, the spray-coated dried hydrogel of the same concentration yielded a mean DHI value of 1.18, a mean SERS intensity of 651.6 counts with a standard deviation of 211.3 and an RSD% of 32.42%. The almost three-fold reduction in the DHI between drop-casting and spray-coating quantitatively confirms the superior spatial homogeneity achieved by spray-coating. While the RSD% of 32.42% remains elevated, the spatial distribution of pixel intensities across the studied map reveals a markedly more uniform surface coverage, with no defined high-intensity core region.

These results collectively demonstrate that spray-coating, even in its non-optimized state, already outperforms drop-casting in terms of the spatial homogeneity of AgNP deposition on the hydrogel surface. The remaining variability, reflected in the RSD% of 32.42%, is consistent with the unoptimized nature of the spray-coating parameters at this stage, and directly motivates the optimization workflow. Full optimization of spray-coating parameters, including AgNP concentration, flow rate, time and distance, is therefore a critical prerequisite for unlocking the full performance potential of the SER-CI method, especially in the biomedical area, such as skin permeation studies.

## 3. Materials and Methods

### 3.1. Chemicals and Reagents

Gelatine from porcine skin (Type A) was purchased from Sigma Aldrich (Saint-Louis, MO, USA). Agarose and diphenhydramine hydrochloride were obtained from Thermo Fischer Scientific (Waltham, MA, USA). Glycerol was purchased from Acros Organics (Geel, Belgium). All solutions were prepared using ultrapure water (18.2 MΩ.cm, Purelab Chorus, ELGA LabWater, Wycombe, UK).

### 3.2. Polymeric Matrix Preparation and Drying

Gelatine–agarose hydrogels were prepared in 4 cm diameter Petri dishes for each experimental condition. First, 15.0 mL of ultrapure water was transferred into a beaker with a glass volumetric pipette and heated to 80 °C under magnetic agitation. Once the temperature was reached, a mixture of gelatine and agarose was added to the heated water at a final concentration of 1.5% (*w*/*v*) for each polymer [63,87]. The mixture was maintained at 80 °C for 15 min under constant stirring to ensure complete dissolution and homogenization. It was then allowed to cool down to 50 °C at room temperature for 5 min, and 5% (*v*/*v*) of glycerol was added and thoroughly mixed to ensure uniform distribution using a magnetic stirrer. Diphenhydramine hydrochloride was then dissolved in the beaker to obtain a concentration of 50 mg·mL^−1^, under magnetic agitation after cooling down, to avoid the heat-induced chemical degradation of the analyte. The mixture was then brought to 20.0 mL using ultrapure water. From each preparation, 1.0 mL of hydrogel was poured into individual glass Petri dishes, prepared in several replicates per condition. The gels were allowed to stabilize at room temperature for 5 min. Finally, the Petri dishes were transferred to an incubator set at 25 °C under controlled relative humidity (RH) condition of 60%. Each dish was loosely covered with a Petri dish lid to allow air exchange, and the gels were left to undergo dehydration.

Hydrogels were produced in three independent batches on three consecutive days (*n* = 3; one batch per day). For each batch, nine gels were cast from the same mixture and served as within-batch subsamples.

### 3.3. Polymeric Matrix Evaluation Strategy

A semi-empirical experimental approach was followed for the evaluation of dehydrated polymeric films. Although no full experimental design or risk mapping was conducted, selected concepts from the ICH Q8 guideline [88] were applied, as the polymeric matrix is intended to support future analytical imaging applications. In this context, the CQAs were defined with the following performance attributes: (i) the polymeric matrix must enable spatially uniform analyte distribution across its exploitable surface and exhibit fingerprint signal-mimicking skin models; (ii) when dried, it must enable the homogeneous distribution of API, without any drying pattern; (iii) it must demonstrate stability over time and exhibit repeatable drying kinetics across intra- and inter-day triplicates; and (iv) the matrix must be compatible with imaging-based analytical techniques such as SER-CI, providing sufficient signal quality and repeatability to validate its intended analytical application.

### 3.4. Analytical Evaluation

Analytical evaluation of the dried hydrogel matrices was carried out to assess the Raman fingerprint common bands compared to a reconstructed human epidermis (RHE) model and the repeatability of preparation and the spatial homogeneity of the analyte signal inside the gel cast. Raman imaging was performed on nine independently prepared gels per day over three experimental days (*n* = 27), enabling the evaluation of intra-gel, intra-batch, and inter-batch variability.

#### 3.4.1. Reconstructed Human Epidermis Model

EpiSkin^®^ reconstructed human epidermis (RHE) models were selected and purchased from EpiSkin^®^ (Lyon, France), and were received as individual inserts in 12-well plates at maturation day 12. Upon arrival, each tissue was transferred from its agarose storage medium into 2 mL of phenol red-free maintenance medium (EpiSkin^®^ Lyon, France) and incubated for a minimum of 18 h at 37 °C, under saturated humidity and 5% CO_2_ atmosphere. On the following day, tissues were subjected to permeation with sterile phosphate-buffered saline (PBS, Lonza, Basel, Switzerland), renewed hourly over an 8 h period. RHE models were then stored at −80 °C overnight. On the next day, tissues were embedded and sectioned transversally at 20 µm using a Leica cryostat (CM3050S, Leica, Wetzlar, Germany), and sections were directly cryo-fixed onto SuperFrost Plus^TM^ glass microscope slides (VWR, Radnor, PA, USA).

#### 3.4.2. Raman Imaging

Raman hyperspectral maps were acquired using a LabRAM HR Evolution Raman microscope (Horiba, Kyoto, Japan) equipped with a 532 nm laser source for the dried hydrogel and a 785 nm laser source to avoid fluorescence phenomenon for the EpiSkin^®^ RHE samples. Both samples were deposited onto SuperFrost Plus^TM^ glass microscope slides (VWR, Radnor, PA, USA) as Raman imaging acquisition substrates. The acquisition parameters for the evaluation are summarized in Table 4. A total analysis time of 1 h was conducted for the individual map acquisition of samples.

#### 3.4.3. Data Treatment

Raman data were processed using MatLab^®^ R2022b (The MathWorks, Natick, MA, USA) in combination with PLS_Toolbox 8.9.2 (Eigenvector Research Inc., Wenatchee, WA, USA). Spectra were baseline-corrected using a Whittaker filter (smoothing parameter λ = 3.0 × 10^4^; asymmetry parameter *p* = 0.001), then inspected to select robust, well-resolved bands for analysis. The most intense diphenhydramine-specific band (1003 cm^−1^) and the glycerol band (1469 cm^−1^) were retained. The following processing approaches were considered: application of a ratiometric correction using $I_{1003{cm}^{-1}}/I_{1469{cm}^{-1}}$ ($I_{ratio}$) to mitigate overall intensity variations (e.g., thickness/focus fluctuations) and track dehydration-induced changes. For each time point, the mean $I_{ratio}$ ± SD and RSD% were computed using pixel intensities across all pixels. Across-replicate RSD% of the normalized signal was used to quantify signal reproducibility.

#### 3.4.4. Distributional Homogeneity Index

Spatial signal homogeneity was quantified using the Distributional Homogeneity Index (DHI), as described by Sacré et al. [75]. For each analyte distribution map, macropixel analysis with a Continuous-Level Moving Block (CLMB) scheme was applied by averaging all possible square blocks of increasing size (2 × 2, 3 × 3, … up to the full image). For each block size s, the standard deviation across all s × s blocks of the corresponding block-mean intensities, ${SD}_{SM}\left(s\right)$, was computed and plotted as a function of s to generate the homogeneity curve. A reference curve, ${SD}_{RM}\left(s\right)$, was obtained from randomized versions of the same map generated by permuting pixel intensities.

The DHI was then calculated as the ratio of areas under the experimental and randomized homogeneity curves (AUCs), as defined in Equation (1):

(1) $$DHI=\bar{\left(\frac{AUC\left({SD}_{SM}\left(s\right)\right)}{AUC\left({SD}_{RM}\left(s\right)\right)}\right)}$$

The DHI calculations were performed in MatLab^®^ R2022b (The MathWorks, Natick, MA, USA). Hyperspectral cubes were first resampled onto a common spatial grid and preprocessed as in Section 3.4.3. Analyte distribution maps were directly normalized by applying the univariate ratio map $\left(I_{ratio}\right)$ for the ratiometric approach.

### 3.5. Physical Evaluation of Drying Kinetics

To assess macroscopic integrity, films were visually inspected for signs of gel cracking or analyte crystallization. A microscopic evaluation was performed using Raman imaging to detect microstructural anomalies, such as cracks, and analyte distribution.

Drying kinetics were monitored gravimetrically by measuring mass loss at defined time intervals during dehydration. Gravimetric water-loss measurements were collected for each gel from T_0h_, defined as the baseline immediately after casting and initial gelation through T_24h_ (24 h post-initiation) at predefined time points. At each time point, gels were weighed and immediately returned to controlled environmental conditions (25 °C, 60% RH). The observation window was limited to 24 h to capture the approach to equilibrium moisture content; analytical evaluations were initiated at the stabilization time point. Accordingly, time points acquired after stabilization were excluded from kinetic analyses.

#### Mixed-Effects Modelling of Drying Kinetics

Water loss was quantified as the relative change in mass overtime, reported as percentage of mass loss, enabling the assessment of drying kinetics as a function of time. Relative water content (RWC) at each time point was computed as per Equation (2) [84]:

(2) $$RWC\;(t)\;=\;\frac{m\left(t\right)\;-\;m_{dry}}{m_{initial}\;-\;m_{dry}}$$

where *m*(*t*) is the measured mass at time *t*, *m_initial_* is the initial mass at T_0h_, and *m_dry_* is the final constant mass after complete drying.

To analyze the kinetics and the repeatability of hydrogel preparation, RWC data were modelled within a linear mixed-effects framework, after a log transformation of the datasets, which accounts for both fixed and random sources of variability. The general structure of the model can be expressed as Equation (3) [89]:

*Y_ijk_* = *μ* + *T_i_* + *P_j_* + *C_k_* + *e_ijk_* (3)

where *Y_ijk_* is the observed RWC at time i for gel j in batch k, µ is the overall mean, *T_i_* is the fixed effect of time in hours (i = 0 to 22 h), *P_j_* is the random effect of gel nested in day component (j = 1 to 9), *C_k_* is the fixed effect of batch for the inter-day variability assessment (k = 1 to 3), and *e_ijk_* is the residual error. This structure is particularly adapted for longitudinal data in which repeated measurements are conducted on the same gels and takes account for the hierarchical nesting of gels within batches (for each day) and the temporal correlation between observations.

Data processing and statistical analyses were performed in JMP Student Edition^®^ 19.0.5 (SAS Institute, Cary, NC, USA). Competing correlation structures were systematically tested to model temporal dependencies in the residuals. Model selection was guided by Akaike’s Information Criterion (AIC) and Bayesian Information Criterion (BIC), with the lowest values indicating the best-fitting structure while avoiding overparameterization [90].

The final selected model was further validated by residual diagnostics to ensure homoscedasticity and the absence of systematic trends. Model-based 95% prediction intervals (PIs) were derived for repeated observations at each time point, where the PI represents the expected gel-to-gel variability. Statistical significance was assessed at α = 0.05 [79].

### 3.6. SER-CI Testing on Dried Hydrogels

Silver nanoparticles (AgNPs) synthetized according to the microwave-assisted protocol of Horne et al. [44] were first concentrated using a centrifuge (Eppendorf, Hamburg, Germany), where the supernatant was removed to obtain a 10-times higher relative concentration. Dried hydrogels were prepared according to Section 3.2, and only the concentration of diphenhydramine hydrochloride from 0.1 to 10 mg·mL^−1^ was tested.

For the spray-coating deposition technique, a lab-made automated device was used, composed of a 500 µL gas tight syringe (Hamilton, Reno, NV, USA), fixed on a Legato^®^ 100 syringe pump (KD scientific, Holliston, MA, USA). The AgNP suspension was pumped through a 100 cm long peek tube from Waters (Milford, MA, USA) with an internal diameter of 0.127 mm and sprayed through an Electrospray Ionization probe (Waters, Milford, USA), placed in a stainless steel capillary tube connected to a nitrogen inlet, used as a spraying gas (Air Liquide, Paris, France). The device was mounted onto a robotic arm from Dobot Robotics (Dobot MG400, Shenzhen, China) automated through an informatically controlled programme. The syringe pump was fixed at 15 µL/min, and the sample was sprayed for 20 min, and the spraying gas was kept at 3 bars to ensure sufficient nebulization. The robotic arm speed was set at 25% of its maximal limit, while keeping the sample at 12 cm from the probe for the experiments. SER-CI analyses were directly conducted, following the same parameters as for dried hydrogels of Section 3.4.2, except for the neutral density (ND) filter that was lowered at 1% to avoid the heat-induced burning of AgNPs due to the powerful laser intensity.

For the drop-casting technique, 20 µL of the 10-times concentrated AgNPs were deposited onto the dried hydrogels with an automated pipette and left to dry before the SER-CI analysis, following the same parameters as those in the spray-coating condition.

## 4. Conclusions

This work establishes a proof of concept for a repeatable, dried hydrogel matrix as a standardized analytical platform for chemical imaging method development and optimization. The matrix was designed, characterized, and validated to meet predefined CQA requirements: spectral skin mimicry ability and spatial uniformity of analyte distribution, controlled and reproducible drying kinetics, and compatibility with Raman effect-associated chemical imaging techniques. Additionally, the dried hydrogel was designed to display a protein-dominated fingerprint consistent with its gelatine matrix composition, evidenced by characteristic collagen-associated bands. These signatures are similarly retrieved in the EpiSkin^®^ RHE SC spectrum, supporting the relevance of the hydrogel as a protein-based skin matrix surrogate.

Drying kinetics were rigorously characterized through mixed-effects modelling, revealing a repeatable process by the diffusion-controlled removal of bound water with stable equilibrium around 22% residual water content. The narrow prediction intervals and absence of inter-day variability confirmed robust process control under fixed conditions (25 °C, 60% RH). Diphenhydramine hydrochloride, selected through compatibility screening (pH, logP, solubility, molecular weight), was successfully incorporated without inducing gelation failure, crystallization, or detectable spatial aggregation.

Analytical evaluation using confocal Raman imaging demonstrated that normalization-based readout ${(I}_{ratio}$) provided precision, with CQAs all within ICH Q8-aligned values [88]. The spatial homogeneity assessment via the Distribution Homogeneity Index yielded values close to 1, confirming homogeneity in analyte distribution. Based on the development dataset, acceptance criteria were refined and validated through an independent confirmation batch. All criteria were met with substantial margins, demonstrating process capability and fit-for-purpose as a calibration standard.

Future work could expand API compatibility screening to establish compatibility specification based on the polymeric structure of the dried hydrogel. Adaptation to complementary chemical imaging modalities by precise tailoring based on limitations, particularly for imaging techniques based on matrix covering such as MALDI-MSI, would validate the utility of the present matrix for future applications. SER-CI method development constitutes the main perspective of this approach, as the tailored model could be used to develop and optimize the sample preparation step of nanoparticle covering for quantitative approaches of SER-CI, as illustrated by the comparison study of drop-casting and spray-coating deposition techniques using the developed dried hydrogel. Spray-coating deposition technique, as also used in the applicative section of this study, could enhance the analytical performances of SER-CI, especially in the biomedical area where the inherent variability of complex matrices could hinder the reproducibility of the method.

By bridging overly simplified synthetic systems and uncontrolled biological variability, this matrix accelerates method optimization, enables robust performance qualification, and could pave the way to the implementation of chemical in routine pharmaceutical and biomedical applications. Continued refinement following the ICH Q8 lifecycle [88] principles will enhance its utility across diverse imaging platforms.

## Figures and Tables

**Table 1:** Normalized intensity ratio $I_{ratio}$ mapping: development set performance. Hyperspectral Raman maps acquired from diphenhydramine hydrochloride-loaded hydrogels across three manufacturing days *(n* = 9 gels per day, 27 total). Intra-day RSDs quantify gel-to-gel variability within each batch; inter-day RSD quantifies day-to-day manufacturing repeatability. DHI was computed via CLMB procedure.

	Day 1	Day 2	Day 3
**Intensity Metrics**			
**Mean** $I_{ratio}$ **(per batch)**	2.67 ± 0.10	2.62 ± 0.20	2.62 ± 0.21
**Intra-day RSD (%)**	3.55	7.64	8.15
**Inter-day RSD (%)**	6.47		
**Spatial Distribution**			
**Mean pixel-wise RSD, intra-day (%)**	8.30 ± 2.79	10.97 ± 3.51	12.33 ± 1.35
**Pixel-wise RSD, inter-day (%)**	10.53		
**Mean DHI (per day)**	1.24	1.14	1.14

**Table 2:** Independent confirmation validation. Three-gel confirmatory batch manufactured from a single batch and analyzed identically to development set.

	Normalization
	Confirmatory batch (*n* = 3)
**Intensity Metrics**	
**Mean** $I_{ratio}$ **(per batch)**	2.60 ± 0.15
**Intra-day RSD (%)**	1.41
**Spatial Distribution**	
**Pixel-wise RSD, intra-day (%)**	9.74
**Mean DHI (per batch)**	1.16

**Table 3:** Fixed-effects parameter estimates from the mixed-effects model describing drying kinetics of hydrogels. Estimates (±standard error) correspond to the log-transformed RWC, as a function of day and time. The t ratio represents the *t*-statistic testing whether each estimates differs significantly from zero. Prob > |t| gives the *p*-value; significant terms (*p* < 0.05) indicate time points that differ from the reference and is highlighted with *.

Term	Estimate	Standard Error	Prob > |t|
Intercept	−1.51	1.36 × 10^−2^	<0.001 *
Batch [Day 1–Day 3]	7.93 × 10^−3^	1.63 × 10^−2^	0.64
Batch [Day 1–Day 2]	9.00 × 10^−3^	1.62 × 10^−2^	0.60
Batch [Day 2–Day 3]	8.62 × 10^−3^	1.62 × 10^−2^	0.95
Time [0 h–22 h]	1.50	1.20 × 10^−2^	<0.001 *
Time [2 h–22 h]	1.39	1.20 × 10^−2^	<0.001 *
Time [4 h–22 h]	1.28	1.20 × 10^−2^	<0.001 *
Time [20 h–22 h]	−1.64 × 10^−3^	1.98 × 10^−3^	0.42
Time [21 h–22 h]	−9.99 × 10^−4^	1.31 × 10^−3^	0.47

**Table 4:** Raman imaging parameters.

	Hydrogels	EpiSkin^®^ RHE
Laser source	532 nm	785 nm
Gratings	300 g/mm	300 g/mm
Spectral resolution	19.84 cm^−1^	8.79 cm^−1^
ND Filter	100%	100%
Spectral range	200–3200 cm^−1^	500–1800 cm^−1^
Objective	MPLAN 10×/0.25 NA	MPLAN 10×/0.25 NA
Confocal hole	200 µm	100 µm
Mapping area	9 mm^2^ composed of 30 × 30 pixels	0.2 mm^2^ composed of 20 × 20 pixels
Step size	300 µm	10 µm
Acquisition	1 s per point, 2 accumulations	15 s per point, 2 accumulations

## Data Availability

Data available on request.

## Supplementary Materials

The following supporting information can be downloaded at: https://www.mdpi.com/article/10.3390/molecules31091530/s1, Figure S1: S/N ratio assessment of the diphenhydramine tracer band. (a) S/N ratio map per pixel computed as peak (995–1010 cm^−1^)/RMS (1800–1850 cm^−1^) on baseline-corrected spectra (25 × 25-pixels). (b) Histogram of S/N ratio per pixel values with a unimodal distribution and mild right trail; Table S1: Results for Day 1 of spatial homogeneity evaluation of dried hydrogels; Table S2: Results for Day 2 of spatial homogeneity evaluation of dried hydrogels; Table S3: Results for Day 3 of spatial homogeneity evaluation of dried hydrogels; Table S4: Results for the confirmatory batch of spatial homogeneity evaluation of dried hydrogels; Table S5: Gravimetric measurements of Day 1, transformed to relative water content in percent, used for mixed and Weibull modelling; Table S6: Gravimetric measurements of Day 2, transformed to relative water content in percent, used for mixed and Weibull modelling; Table S7: Gravimetric measurements of Day 3, transformed to relative water content in percent, used for mixed and Weibull modelling; Equation (S1): Bimodal Weibull modelling equation of RWC prediction; Figure S2: Bimodal Weibull fit for hydrogel drying kinetics; Table S8: Estimated parameters of the bimodal Weibull model and the corresponding performances metrics; Figure S3: Residuals versus fitted values for the bimodal no-lag Weibull model; Table S9: Akaike and Bayesian Information Criteria for alternative within-gel covariance structures; Figure S4: Model adequacy diagnostic for the mixed-effect model by QQ-plot; Table S10: Data table of mean SER-CI intensity, standard deviation and relative standard deviation from analysis of dried hydrogels containing different concentrations of diphenhydramine hydrochloride, from 0.1 to 10 mg L^−1^.

## Abbreviations

The following abbreviations are used in this manuscript:

AgNPs	Silver nanoparticles
AIC	Akaike’s Information Criterion
ANTE-EV	Antedependence equal variance model
API	Active pharmaceutical ingredient
AUC	Area Under the Curve
BIC	Bayesian Information Criterion
CLMB	Continuous-Level Moving Block
CQA	Critical Quality Attribute
DHI	Distributional Homogeneity Index
EMA	European Medicines Agency
ICH	International Council for Harmonization
I_ratio_	Intensity after ratiometric normalization of $I_{1003\;{cm}^{-1}}/I_{1469\;{cm}^{-1}}$
IR	Infra-red
MALDI-MSI	Matrix-Assisted Laser Desorption Ionization Mass Spectrometry Imaging
ND	Neutral density
NP	Nanoparticle
OECD	Organisation for Economic Co-operation and Development
PBS	Phosphate-buffered saline
PI	Prediction interval
Prob	Probability
R-CI	Raman chemical imaging
RH	Relative humidity
RHE	Reconstructed human epidermis
RSD	Relative standard deviation
RWC	Relative water content
SC	Stratum corneum
SER-CI	Surface-Enhanced Raman Chemical Imaging
SNR	Signal-to-noise ratio

## References

[1] Smith G.P.S., McGoverin C.M., Fraser S.J., Gordon K.C. (2015). Raman imaging of drug delivery systems. Adv. Drug Deliv. Rev..

[2] Kichou H., Bonnier F., Dancik Y., Bakar J., Michael-Jubeli R., Caritá A.C., Perse X., Soucé M., Rapetti L., Tfayli A. (2023). Strat-M^®^ positioning for skin permeation studies: A comparative study including EpiSkin^®^ RHE, and human skin. Int. J. Pharm..

[3] OECD (2004). Test No. 428: Skin Absorption: In Vitro Method.

[4] OECD (2004). Guidance Document for the Conduct of Skin Absorption Studies.

[5] Quality and Equivalence of Locally Applied, Locally Acting Cutaneous Products—Scientific Guideline|European Medicines Agency (EMA). https://www.ema.europa.eu/en/quality-equivalence-locally-applied-locally-acting-cutaneous-products-scientific-guideline.

[6] Orkoula M.G., Kontoyannis C.G., Markopoulou C.K., Koundourellis J.E. (2006). Quantitative analysis of liquid formulations using FT-Raman spectroscopy and HPLC The case of diphenhydramine hydrochloride in Benadryl. J. Pharm. Biomed. Anal..

[7] Kichou H., Munnier E., Dancik Y., Kemel K., Byrne H., Tfayli A., Bertrand D., Soucé M., Chourpa I., Bonnier F. (2022). Estimating the Analytical Performance of Raman Spectroscopy for Quantification of Active Ingredients in Human Stratum Corneum. Molecules.

[8] Bielfeldt S., Bonnier F., Byrne H.J., Chourpa I., Dancik Y., Lane M.E., Lunter D.J., Munnier E., Puppels G., Tfayli A. (2022). Monitoring dermal penetration and permeation kinetics of topical products; the role of Raman microspectroscopy. TrAC Trends Anal. Chem..

[9] Lohumi S., Kim M.S., Qin J., Cho B.-K. (2017). Raman imaging from microscopy to macroscopy: Quality and safety control of biological materials. TrAC Trends Anal. Chem..

[10] Zsikó S., Csányi E., Kovács A., Budai-Szűcs M., Gácsi A., Berkó S. (2019). Methods to Evaluate Skin Penetration In Vitro. Sci. Pharm..

[11] Binder L., Kulovits E.M., Petz R., Ruthofer J., Baurecht D., Klang V., Valenta C. (2018). Penetration monitoring of drugs and additives by ATR-FTIR spectroscopy/tape stripping and confocal Raman spectroscopy—A comparative study. Eur. J. Pharm. Biopharm..

[12] Eyer K., Paech F., Schuler F., Kuhn P., Kissner R., Belli S., Dittrich P.S., Krämer S.D. (2014). A liposomal fluorescence assay to study permeation kinetics of drug-like weak bases across the lipid bilayer. J. Control. Release.

[13] Schmälzlin E., Moralejo B., Gersonde I., Schleusener J., Darvin M.E., Thiede G., Roth M.M. (2018). Nonscanning large-area Raman imaging for ex vivo/in vivo skin cancer discrimination. JBO.

[14] Egawa M. (2021). Raman microscopy for skin evaluation. Analyst.

[15] Essendoubi M., Alsamad F., Noël P., Meunier M., Scandolera A., Sandré J., Manfait M., Gobinet C., Reynaud R., Piot O. (2021). Combining Raman imaging and MCR-ALS analysis for monitoring retinol permeation in human skin. Skin Res. Technol..

[16] Zeng Q., Wang L., Wu S., Fang G., Zhao M., Li Z., Li W. (2022). Research progress on the application of spectral imaging technology in pharmaceutical tablet analysis. Int. J. Pharm..

[17] Kichou H., Bonnier F., Caritá A., Byrne H., Chourpa I., Munnier E. (2024). Confocal Raman spectroscopy coupled with in vitro permeation testing to study the effects of formalin fixation on the skin barrier function of reconstructed human epidermis. Spectrochim. Acta Part A Mol. Biomol. Spectrosc..

[18] Bonnel D., Legouffe R., Willand N., Baulard A., Hamm G., Deprez B., Stauber J. (2011). MALDI imaging techniques dedicated to drug-distribution studies. Bioanalysis.

[19] Mirnezami R., Spagou K., Vorkas P.A., Lewis M.R., Kinross J., Want E., Shion H., Goldin R.D., Darzi A., Takats Z. (2014). Chemical mapping of the colorectal cancer microenvironment via MALDI imaging mass spectrometry (MALDI-MSI) reveals novel cancer-associated field effects. Mol. Oncol..

[20] Horgan C.C., Jensen M., Nagelkerke A., St-Pierre J.-P., Vercauteren T., Stevens M.M., Bergholt M.S. (2021). High-Throughput Molecular Imaging via Deep-Learning-Enabled Raman Spectroscopy. Anal. Chem..

[21] Dunnington E.L., Wong B.S., Fu D. (2024). Innovative Approaches for Drug Discovery: Quantifying Drug Distribution and Response with Raman Imaging. Anal. Chem..

[22] Tfayli A., Piot O., Pitre F., Manfait M. (2007). Follow-up of drug permeation through excised human skin with confocal Raman microspectroscopy. Eur. Biophys. J..

[23] Brozek-Pluska B. (2020). Statistics assisted analysis of Raman spectra and imaging of human colon cell lines—Label free, spectroscopic diagnostics of colorectal cancer. J. Mol. Struct..

[24] Bakonyi M., Gácsi A., Kovács A., Szűcs M.-B., Berkó S., Csányi E. (2018). Following-up skin penetration of lidocaine from different vehicles by Raman spectroscopic mapping. J. Pharm. Biomed. Anal..

[25] Bookmeyer C.H.M., Correig F.X., Masana L., Magni P., Yanes Ó., Vinaixa M. (2025). Advancing atherosclerosis research: The Power of lipid imaging with MALDI-MSI. Atherosclerosis.

[26] Hu J.-B., Chen Y.-C., Urban P.L. (2013). Coffee-ring effects in laser desorption/ionization mass spectrometry. Anal. Chim. Acta.

[27] Gautier J., Allard-Vannier E., Hervé-Aubert K., Soucé M., Chourpa I. (2013). Design strategies of hybrid metallic nanoparticles for theragnostic applications. Nanotechnology.

[28] Bock S., Choi Y.-S., Kim M., Yun Y., Pham X.-H., Kim J., Seong B., Kim W., Jo A., Ham K.-M. (2022). Highly sensitive near-infrared SERS nanoprobes for in vivo imaging using gold-assembled silica nanoparticles with controllable nanogaps. J. Nanobiotechnol..

[29] Du Z., Qi Y., He J., Zhong D., Zhou M. (2021). Recent advances in applications of nanoparticles in SERS in vivo imaging. WIREs Nanomed. Nanobiotechnol..

[30] Wang Y.W., Doerksen J.D., Kang S., Walsh D., Yang Q., Hong D., Liu J.T.C. (2016). Multiplexed molecular imaging of fresh tissue surfaces enabled by convection-enhanced topical staining with SERS-coded nanoparticles. Small.

[31] Huo C., Han W., Tang W., Duan X. (2021). Stable SERS substrate based on highly reflective metal liquid-like films wrapped hydrogels for direct determination of small molecules in a high protein matrix. Talanta.

[32] Ilișanu M.-A., Moldoveanu F., Moldoveanu A. (2023). Multispectral Imaging for Skin Diseases Assessment—State of the Art and Perspectives. Sensors.

[33] Qiu C., Zhang W., Zhou Y., Cui H., Xing Y., Yu F., Wang R. (2023). Highly sensitive surface-enhanced Raman scattering (SERS) imaging for phenotypic diagnosis and therapeutic evaluation of breast cancer. Chem. Eng. J..

[34] Qiu C., Cheng Z., Lv C., Wang R., Yu F. (2021). Development of bioorthogonal SERS imaging probe in biological and biomedical applications. Chin. Chem. Lett..

[35] Nicolae-Maranciuc A., Chicea D. (2025). Polymeric Systems as Hydrogels and Membranes Containing Silver Nanoparticles for Biomedical and Food Applications: Recent Approaches and Perspectives. Gels.

[36] Goudie K.J., McCreath S.J., Parkinson J.A., Davidson C.M., Liggat J.J. (2023). Investigation of the influence of pH on the properties and morphology of gelatin hydrogels. J. Polym. Sci..

[37] Kozuka H. (2018). Stress Evolution and Cracking in Sol-Gel-Derived Thin Films. Handbook of Sol-Gel Science and Technology.

[38] Waje S.S., Meshram M.W., Chaudhary V., Pandey R., Mahanawar P.A., Thorat B.N. (2005). Drying and shrinkage of polymer gels. Braz. J. Chem. Eng..

[39] Šimáková P., Kočišová E., Procházka M. (2021). “Coffee Ring” Effect of Ag Colloidal Nanoparticles Dried on Glass: Impact to Surface-Enhanced Raman Scattering (SERS). J. Nanomater..

[40] Mampallil D., Eral H.B. (2018). A review on suppression and utilization of the coffee-ring effect. Adv. Colloid Interface Sci..

[41] Cailletaud J., De Bleye C., Dumont E., Sacré P.-Y., Gut Y., Bultel L., Ginot Y.-M., Hubert P., Ziemons E. (2018). Towards a spray-coating method for the detection of low-dose compounds in pharmaceutical tablets using surface-enhanced Raman chemical imaging (SER-CI). Talanta.

[42] De Bleye C., Fontaine M., Dumont E., Sacré P.-Y., Hubert P., Theys N., Ziemons E. (2020). Raman imaging as a new analytical tool for the quality control of the monitoring of osteogenic differentiation in forming 3D bone tissue. J. Pharm. Biomed. Anal..

[43] Horne J., De Bleye C., Lebrun P., Kemik K., Van Laethem T., Sacré P.-Y., Hubert P., Hubert C., Ziemons E. (2023). Optimization of silver nanoparticles synthesis by chemical reduction to enhance SERS quantitative performances: Early characterization using the quality by design approach. J. Pharm. Biomed. Anal..

[44] Horne J., Beckers P., Sacré P.-Y., De Bleye C., Francotte P., Thelen N., Hubert P., Ziemons E., Hubert C. (2024). Optimisation of a Microwave Synthesis of Silver Nanoparticles by a Quality by Design Approach to Improve SERS Analytical Performances. Molecules.

[45] Barani H., Mahltig B. (2022). Microwave-Assisted Synthesis of Silver Nanoparticles: Effect of Reaction Temperature and Precursor Concentration on Fluorescent Property. J. Clust. Sci..

[46] Cao J., Hu S., Tang W., Wang Y., Yang Y., Wang F., Guo X., Ying Y., Liu X., Wen Y. (2023). Reactive Hydrogel Patch for SERS Detection of Environmental Formaldehyde. ACS Sens..

[47] Dumont E., De Bleye C., Rademaker G., Coïc L., Horne J., Sacré P.-Y., Peulen O., Hubert P., Ziemons E. (2021). Development of a prototype device for near real-time surface-enhanced Raman scattering monitoring of biological samples. Talanta.

[48] Matsumoto C., Gen M., Matsuki A., Seto T. (2022). Development of spray-drying-based surface-enhanced Raman spectroscopy. Sci. Rep..

[49] Bickerstaff-Westbrook E., Tukova A., Lyu N., Shen C., Rodger A., Wang Y. (2024). Advancing SERS label-free detection of bacteria: Sensing in liquid vs drop-cast. Mater. Today Sustain..

[50] Avci E., Culha M. (2013). Influence of droplet drying configuration on surface-enhanced Raman scattering performance. RSC Adv..

[51] De Bleye C., Sacré P.-Y., Dumont E., Netchacovitch L., Chavez P.-F., Piel G., Lebrun P., Hubert P., Ziemons E. (2014). Development of a quantitative approach using surface-enhanced Raman chemical imaging: First step for the determination of an impurity in a pharmaceutical model. J. Pharm. Biomed. Anal..

[52] Shaikh I., Sartale S. (2023). Spin coated Ag NPs SERS substrate: Trace detection study of methylene blue and melamine. Appl. Phys. A.

[53] Mikalkevičius M., Khinevich N., Tamulevičius S., Tamulevičius T., Tamulevičienė A. (2024). Templated silver nanoparticle deposition on laser-induced periodic surface structures for SERS sensing. Surf. Interfaces.

[54] Wang S., Wang Z., Cao X., Wang G., Guo R., Zewdie Y., Li S., Zhang L., Dong Q., Chen Z. (2025). Uniform Spray-Coated Flexible SERS Substrates for Enhanced Molecular Detection. Chem. Asian J..

[55] Boel E., Koekoekx R., Dedroog S., Babkin I., Vetrano M.R., Clasen C., Van den Mooter G. (2020). Unraveling Particle Formation: From Single Droplet Drying to Spray Drying and Electrospraying. Pharmaceutics.

[56] Zheng X.-S., Jahn I.J., Weber K., Cialla-May D., Popp J. (2018). Label-free SERS in biological and biomedical applications: Recent progress, current challenges and opportunities. Spectrochim. Acta Part A Mol. Biomol. Spectrosc..

[57] Moody A.S., Payne T.D., Barth B.A., Sharma B. (2020). Surface-enhanced spatially-offset Raman spectroscopy (SESORS) for detection of neurochemicals through the skull at physiologically relevant concentrations. Analyst.

[58] Jepps O.G., Dancik Y., Anissimov Y.G., Roberts M.S. (2013). Modeling the human skin barrier—Towards a better understanding of dermal absorption. Adv. Drug Deliv. Rev..

[59] Dąbrowska A.K., Rotaru G.-M., Derler S., Spano F., Camenzind M., Annaheim S., Stämpfli R., Schmid M., Rossi R.M. (2016). Materials used to simulate physical properties of human skin. Skin Res. Technol..

[60] Raj P., Wu L., Arora S., Bhatt R., Zuo Y., Fang Z., Verdoold R., Koch T., Gu L., Barman I. (2024). Engineering vascularized skin-mimetic phantom for non-invasive Raman spectroscopy. Sens. Actuators B Chem..

[61] Ntombela L., Adeleye B., Chetty N. (2020). Low-cost fabrication of optical tissue phantoms for use in biomedical imaging. Heliyon.

[62] Nunekpeku X., Li H., Zahid A., Li C., Zhang W. (2025). Advances in Hydrogel-Integrated SERS Platforms: Innovations, Applications, Challenges, and Future Prospects in Food Safety Detection. Biosensors.

[63] Imani R., Emami S.H., Moshtagh P.R., Baheiraei N., Sharifi A.M. (2012). Preparation and Characterization of Agarose-Gelatin Blend Hydrogels as a Cell Encapsulation Matrix: An In-Vitro Study. J. Macromol. Sci. Part B.

[64] Vardaki M.Z., Kourkoumelis N. (2020). Tissue Phantoms for Biomedical Applications in Raman Spectroscopy: A Review. Biomed. Eng. Comput. Biol..

[65] Li J., Mooney D.J. (2016). Designing hydrogels for controlled drug delivery. Nat. Rev. Mater..

[66] Jiang F., Xu X.-W., Chen F.-Q., Weng H.-F., Chen J., Ru Y., Xiao Q., Xiao A.-F. (2023). Extraction, Modification and Biomedical Application of Agarose Hydrogels: A Review. Mar. Drugs.

[67] Diphenhydramine Hydrochloride—European Pharmacopoeia 11.7. https://pheur.edqm.eu/app/11-7/content/11-7/0023E.htm?highlight=on&terms%5B%5D=diphenhydramine.

[68] GMIA Gelatin Manual 2012 | PDF | Cooking, Food & Wine. https://www.scribd.com/doc/148257496/GMIA-Gelatin-Manual-2012.

[69] Xiong J.-Y., Narayanan J., Liu X.-Y., Chong T.K., Chen S.B., Chung T.-S. (2005). Topology evolution and gelation mechanism of agarose gel. J. Phys. Chem. B.

[70] Frushour B.G., Koenig J.L. (1975). Raman scattering of collagen, gelatin, and elastin. Biopolymers.

[71] Payne K.J., Veis A. (1988). Fourier transform IR spectroscopy of collagen and gelatin solutions: Deconvolution of the amide I band for conformational studies. Biopolymers.

[72] Vanin F.M., Sobral P.J.A., Menegalli F.C., Carvalho R.A., Habitante A.M.Q.B. (2005). Effects of plasticizers and their concentrations on thermal and functional properties of gelatin-based films. Food Hydrocoll..

[73] Movasaghi Z., Rehman S., Rehman I.U. (2007). Raman Spectroscopy of Biological Tissues. Appl. Spectrosc. Rev..

[74] Iramain M., Brandán S. (2018). Structural and vibrational properties of three species of anti-histaminic diphenhydramine by using DFT calculations and the SQM approach. Chem. J..

[75] Sacré P.-Y., Lebrun P., Chavez P.-F., Bleye C.D., Netchacovitch L., Rozet E., Klinkenberg R., Streel B., Hubert P., Ziemons E. (2014). A new criterion to assess distributional homogeneity in hyperspectral images of solid pharmaceutical dosage forms. Anal. Chim. Acta.

[76] Smith E., Dent G. (2005). Modern Raman Spectroscopy: A Practical Approach.

[77] Fikry M., Benjakul S., Al-Ghamdi S., Mittal A., Nilsuwan K., Fulleros R., Dabbour M. (2023). Sorption Isotherms and Thermodynamic Characteristics of Gelatin Powder Extracted from Whitefish Skin: Mathematical Modeling Approach. Foods.

[78] Etzold M.A., Linden P.F., Worster M.G. (2021). Transpiration through hydrogels. J. Fluid Mech..

[79] Hummel R., Claassen E.A., Wolfinger R.D. (2021). JMP for Mixed Models.

[80] Adnađević B., Janković B., Kolar-Anić ǈ., Minić D. (2007). Normalized Weibull distribution function for modelling the kinetics of non-isothermal dehydration of equilibrium swollen poly(acrylic acid) hydrogel. Chem. Eng. J..

[81] Janković B., Adnađević B., Jovanović J. (2011). The comparative kinetic study of non-isothermal and isothermal dehydration of swollen poly(acrylic acid) hydrogel using the Weibull probability function. Chem. Eng. Res. Des..

[82] Corzo O., Bracho N., Pereira A., Vásquez A. (2008). Weibull distribution for modeling air drying of coroba slices. LWT—Food Sci. Technol..

[83] Zhang X., Fan Z., Wu H., Cong J., Yang J., Wen B. (2025). Drying characteristics of green pellets based on the Weibull and Dincer models. J. Saf. Sustain..

[84] Torki-Harchegani M., Ghanbarian D., Sadeghi M. (2015). Estimation of whole lemon mass transfer parameters during hot air drying using different modelling methods. Heat Mass Transf..

[85] Hasan M.M.M., Ara R., Shaha L.C., Sarkar A., Alam M. (2025). Modeling the drying behavior and mass transfer phenomena in osmotically dehydrated tomatoes. Food Chem. Adv..

[86] Sánchez-Ferrer A., Engelhardt M., Richter K. (2023). Anisotropic wood–water interactions determined by gravimetric vapor sorption experiments. Cellulose.

[87] Hacker L., Wabnitz H., Pifferi A., Pfefer T.J., Pogue B.W., Bohndiek S.E. (2022). Criteria for the design of tissue-mimicking phantoms for the standardization of biophotonic instrumentation. Nat. Biomed. Eng..

[88] ICH Q8 (R2) Pharmaceutical Development—Scientific Guideline|European Medicines Agency (EMA). https://www.ema.europa.eu/en/ich-q8-r2-pharmaceutical-development-scientific-guideline.

[89] Adutwum J.O., Sakagami H., Koga S., Matsumura J. (2023). An application of mixed-effects model to evaluate the role of age and size on radial variation in wood specific gravity in teak (*Tectona grandis*). J. Wood Sci..

[90] Burnham K.P., Anderson D.R. (2004). Multimodel Inference: Understanding AIC and BIC in Model Selection. Sociol. Methods Res..

