# Supplementary material for: Proof of Concept for a Controlled Raman-Compatible Skin-Mimicking Hydrogel Substrate for Chemical Imaging Technique Development
Source: Molecules. 2026 May 5;31(9):1530. doi: 10.3390/molecules31091530 (PMC13164873; doi:10.3390/molecules31091530)
Supplement: Supplementary file 1 [file molecules-31-01530-s001.zip › molecules-4279325-supplementary.pdf]

Proof of concept for a controlled Raman-compatible skin-mimicking hydrogel substrate for chemical imaging technique development

Kevser Kemik [1,*], Charlotte De Bleye [1], Pierre-Yves Sacré [2], Philippe Hubert [1] and Eric Ziémons [1,*]

[1] Laboratory of Pharmaceutical Analytical Chemistry, ViBra-Santé HUB, CIRM, Department of Pharmacy, University of Liege (ULiege), Avenue Hippocrate 15, 4000 Liège, Belgium; cdebleye@uliege.be (C.D.B.); ph.hubert@uliege.be (P.H.)

[2] Research Support Unit in Chemometrics, CIRM, Department of Pharmacy, University of Liege (ULiege), Avenue Hippocrate, 15, 4000 Liège, Belgium; pysacre@uliege.be

* Correspondence: kevser.kemik@uliege.be (K.K.); eziemons@uliege.be (E.Z.)

Supplementary data

Supplementary note S1. Diphenhydramine signal-to-noise ratio definition

A signal was defined as the peak height within 995-1010 cm^{-1}; noise was expressed as the root mean square (RMS) in a feature-free window, between 1800 and 1850 cm^{-1}, from the same spectrum. The S/N ratio from the mean spectrum for the global sensitivity and the S/N ratio computed per pixel for the map-level robustness were calculated. Under these conditions, the mean-spectrum S/N was determined as 399.97, indicating a strong analytical response at the tracer band. The pixel-wise S/N distribution, as shown on Figure S1 (b), indicates a median S/N of 220.94, with a modest right-hand tailing, consistent with minor local variations rather than matrix interference as can be confirmed on the associated S/N map. The latter shows high S/N values broadly distributed across the field of view, supporting the suitability of the tracer. Observed values are above the usual specification criteria, mainly because the working concentration range is typically higher than the usual concentration ranges, making the Raman signal intense and excluding sensitivity issues.

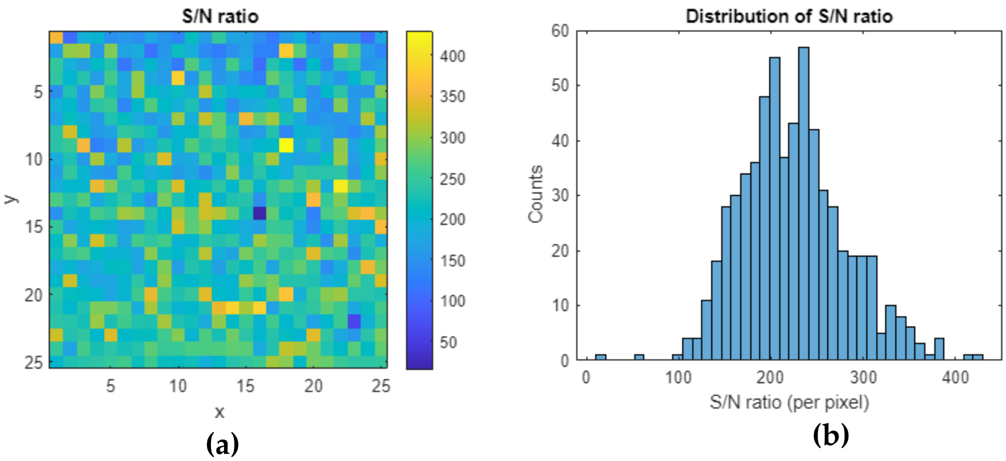

Figure S1. S/N ratio assessment for the diphenhydramine tracer band. (a) S/N map per pixel computed as peak (995-1010 cm^{-1})/RMS (1800-1850 cm^{-1}) on baseline-corrected spectra (25x25-pixels). (b) Histogram of S/N per pixel values with a unimodal distribution and mild right trail.

Supplementary note S2. Raman imaging data

The obtained mean map normalization intensity, RSD (%) and DHI values are provided in Table S1, Table S2 and Table S3 for the three days of the studies, respectively (n = 27), as well as the confirmatory batch (n = 3) in Table S4.

Table S1. Results for Day 1 of spatial homogeneity evaluation of dried hydrogels.

Day 1	Mean normalization intensity	RSD (%)	DHI
1	2.67	8.55	1.12
2	2.79	4.59	1.32
3	2.59	9.82	1.34
4	2.73	8.59	1.32
5	2.74	6.03	1.13
6	2.73	7.13	1.31
7	2.73	6.25	1.19

8	2.58	9.69	1.16
9	2.50	14.06	1.23

Table S2. Results for Day 2 of spatial homogeneity evaluation of dried hydrogels.

Day 2	Mean normalization intensity	RSD (%)	DHI
1	2.58	13.95	1.12
2	2.22	14.21	1.17
3	2.53	14.53	1.29
4	2.55	10.66	1.13
5	2.55	11.84	1.23
6	2.88	3.85	1.02
7	2.70	11.69	1.20
8	2.77	10.88	1.26
9	2.82	7.15	0.88

Table S3. Results for Day 3 of spatial homogeneity evaluation of dried hydrogels.

Day 3	Mean normalization intensity	RSD (%)	DHI
1	2.74	9.70	1.09
2	2.24	11.34	1.08
3	2.24	13.35	1.04
4	2.79	12.41	1.36
5	2.78	11.82	0.96
6	2.69	14.17	1.34
7	2.76	13.73	1.28
8	2.34	12.11	1.12
9	2.74	12.35	1.03

Table S4. Results for the confirmatory batch of spatial homogeneity evaluation of dried hydrogels.

Confirmatory batch	Mean normalization intensity	RSD (%)	DHI
1	2.74	9.05	1.13
2	2.47	11.95	1.12
3	2.60	8.23	1.24

Supplementary note S3. Gravimetric water-loss profiles

The prepared hydrogels were subjected to gravimetric measures every hour to track drying kinetics over three experimental days on Table S5, Table S6 and Table S7, respectively.

Table S5. Gravimetric measurements of Day 1, transformed to relative water content in percent, used for mixed and Weibull modelling.

Time (hours)	Replicate 1	Replicate 2	Replicate 3	Replicate 4	Replicate 5	Replicate 6	Replicate 7	Replicate 8	Replicate 9
0	100.0	100.0	100.0	100.0	100.0	100.0	100.0	100.0	100.0
1	89.28	90.29	90.40	90.49	89.44	90.85	88.41	90.57	92.00
2	78.98	81.49	82.88	82.15	79.48	80.43	78.10	80.80	83.18
3	68.44	72.09	76.27	74.12	67.79	69.29	67.57	70.82	73.04

4	61.78	64.56	69.81	67.84	60.48	60.84	61.47	65.16	66.73
5	52.4	54.63	60.06	58.77	50.77	52.05	53.67	57.47	59.99
6	44.48	46.38	52.19	50.68	42.85	43.95	47.05	50.23	52.44
7	37.84	38.83	44.47	43.23	36.73	34.58	40.68	43.71	45.81
8	32.86	33.22	38.23	37.34	31.92	30.33	35.74	38.24	40.34
9	28.29	28.25	32.22	31.87	27.29	28.14	31.01	32.77	35.28
10	23.99	27.58	27.93	28.18	24.75	25.89	27.79	27.98	31.36
11	23.79	26.78	27.45	27.65	23.85	24.76	25.98	25.61	27.50
12	23.57	26.12	26.56	26.89	23.49	24.15	24.94	24.57	25.04
13	23.36	25.58	26.18	26.28	23.31	24.00	24.54	24.14	24.35
14	23.15	24.55	25.64	25.55	23.14	23.69	23.96	23.72	23.73
15	22.95	24.55	25.02	24.98	23.02	23.38	23.67	23.33	22.91
16	22.72	24.13	24.58	24.45	22.97	23.15	23.49	23.11	22.84
17	22.67	23.65	24.19	24.10	22.79	22.87	23.22	22.92	22.72
18	22.60	23.29	23.7	23.74	22.67	22.62	22.92	22.67	22.62
19	22.51	23.43	23.33	23.28	22.55	22.39	22.59	22.43	22.51
20	22.47	22.40	22.73	22.67	22.47	22.27	22.38	22.34	22.39
21	22.49	22.41	22.38	22.41	22.37	22.19	22.22	22.27	22.28
22	22.48	22.42	22.39	22.43	22.33	21.96	21.60	22.62	22.25

Table S6. Gravimetric measurements of Day 2, transformed to relative water content in percent, used for mixed and Weibull modelling.

Time (hours)	Replicate 1	Replicate 2	Replicate 3	Replicate 4	Replicate 5	Replicate 6	Replicate 7	Replicate 8	Replicate 9
0	100.0	100.0	100.0	100.0	100.0	100.0	100.0	100.0	100.0
1	89.64	88.48	90.63	90.84	89.59	90.40	91.76	92.02	91.57
2	79.54	77.49	81.54	82.31	79.81	81.21	83.45	83.80	83.15
3	69.57	66.48	72.11	73.31	70.13	71.79	74.69	75.38	74.47
4	59.76	56.18	63.24	64.66	60.28	62.69	66.22	67.57	65.79
5	51.10	47.30	55.57	57.00	51.56	54.71	58.57	60.37	55.29
6	43.60	40.06	48.95	50.17	43.55	47.57	51.54	53.78	50.98
7	33.72	32.99	41.89	42.72	36.14	40.38	44.65	46.65	43.92
8	30.97	27.88	35.83	36.31	30.45	34.44	38.38	40.17	37.61
9	27.77	25.27	31.50	31.88	26.86	30.44	33.92	35.43	33.05
10	25.27	25.07	27.88	27.93	24.36	26.89	29.41	30.80	28.82
11	24.97	24.86	25.36	24.98	24.19	25.13	26.85	27.02	26.13
12	24.56	24.64	24.78	24.64	24.05	24.56	25.22	25.43	25.43
13	24.14	24.22	24.33	24.29	23.89	24.19	24.61	25.01	25.00
14	23.78	23.86	23.91	24.02	23.83	23.88	24.22	24.60	24.73
15	23.57	23.61	23.70	23.73	23.71	23.68	23.90	24.36	24.36
16	23.31	23.42	23.47	23.44	23.56	23.47	23.71	24.07	24.03
17	23.04	23.19	23.32	23.22	23.35	23.21	23.49	23.75	23.67
18	22.80	23.10	23.19	23.07	23.17	23.08	23.23	23.44	23.33
19	22.62	22.83	23.07	22.91	22.98	22.96	23.01	23.05	23.07
20	22.41	22.75	23.01	22.77	22.74	22.77	22.78	22.74	22.79
21	22.48	22.82	22.90	22.90	22.80	22.82	22.83	22.79	22.83
22	22.53	22.86	22.85	22.86	22.78	22.86	22.87	22.81	22.86

Table S7. Gravimetric measurements of Day 3, transformed to relative water content in percent, used for mixed and Weibull modelling.

Time (hours)	Replicate 1	Replicate 2	Replicate 3	Replicate 4	Replicate 5	Replicate 6	Replicate 7	Replicate 8	Replicate 9
0	100.0	100.0	100.0	100.0	100.0	100.0	100.0	100.0	100.0
1	88.60	89.32	88.79	88.60	90.20	90.47	90.14	90.64	92.12
2	79.65	79.93	79.90	80.80	82.92	82.66	83.52	83.54	85.79
3	70.15	70.70	70.45	72.70	74.98	74.20	75.54	75.75	76.23
4	60.55	61.18	61.11	64.66	67.74	66.45	67.14	68.68	69.92
5	51.78	49.73	52.02	57.52	60.81	59.08	60.37	61.35	62.41
6	43.32	45.20	43.98	50.56	53.70	51.68	54.12	54.00	55.25
7	38.30	38.90	37.36	44.16	47.19	44.96	46.94	46.85	48.99
8	32.51	33.27	31.71	37.52	41.19	38.88	40.54	40.51	40.06
9	30.64	28.76	27.75	32.57	35.56	33.48	35.22	35.37	35.88
10	26.51	24.85	24.79	28.42	30.40	28.92	30.55	30.74	30.89
11	25.34	24.55	24.60	25.06	26.26	26.12	27.65	27.55	26.86
12	24.65	24.33	24.39	24.65	24.66	24.56	24.76	24.99	24.94
13	24.01	24.04	24.12	24.00	24.15	24.11	24.34	24.45	24.26
14	23.72	23.75	23.81	23.51	23.90	23.88	23.99	24.12	24.01
15	23.36	23.39	23.42	23.15	23.67	23.61	23.72	23.78	23.86
16	22.97	22.94	23.03	22.76	23.10	23.12	23.17	23.23	23.27
17	22.64	22.59	22.64	22.41	22.89	22.74	22.81	23.01	22.84
18	22.38	22.31	22.29	21.97	22.53	22.40	22.53	22.74	22.56
19	22.10	22.03	22.00	21.68	22.10	22.14	22.20	22.24	22.15
20	21.79	21.85	21.72	21.36	21.85	21.93	21.89	21.85	21.81
21	21.83	21.89	21.81	21.17	21.86	21.96	21.92	21.87	21.82
22	21.78	21.85	21.85	21.32	21.19	21.19	21.88	21.83	21.78

Supplementary note S4. Bimodal Weibull model

The Weibull model is widely used as a compact empirical function to describe thin-layer drying and food dehydration kinetics [1-4]. Here, experimental relative water content (RWC) profiles obtained from gravimetric measurements were fitted with the bimodal Weibull function, to parameterize the two-phase drying behaviour of the polymeric analytical matrices. This application remains less commonly reported than in food systems [5-6]. Water loss during controlled drying was modelled according to Eq. 1 [7]:

$$y(t) = b - A_1 \left[1 - \exp\left(-\left(\frac{t}{\tau_1}\right)^{\beta_1}\right)\right] - A_2 \left[1 - \exp\left(-\left(\frac{t}{\tau_2}\right)^{\beta_2}\right)\right] \tag{1}$$

where y(t) is the residual water content (%), b is the initial water content at t=0, A_1 and A_2 are the amplitudes of fast and slow drying phases, τ_1 and τ_2 are characteristic time constants (hours), and β_1 and β_2 are dimensionless shape parameter controlling the curvature of each phase of the decay. This formulation allows a non-zero asymptote, $y(\infty) = b - A_1 - A_2$, consistent with the incomplete dehydration of hydrogels retaining a fraction of bound water.

Fits were performed by nonlinear least squares using MatLab® R2022b, nlinfit and npredici functions; the 95% prediction intervals were calculated using time-dependent residual standard deviation to account for gel-to-gel variability, and goodness-of-fit was assessed using R^2, RMSE, and

corrected AIC (AICc). For pooled analyses, a single set of parameters (b, A_1, A_2, τ_1, τ_2, β_1 and β_2) was estimated across all datasets (n = 27) simultaneously. Residuals were inspected to identify systematic trends indicative of model inadequacy.

The prepared hydrogels were subjected to gravimetric measures every hour to track drying kinetics over the three experimental days. Based on these data (cfr Supplementary note S3), a bimodal Weibull model applied to drying kinetics modelling yielded a close correspondence between the predicted and the experimental data, with a global R^2 around 0.990, as illustrated in Table S8. The RMSE of 2.59% showed a low estimation error of the whole model, capturing the drying curve accurately. Corrected Akaike Information Criterion (AICc) was used to select models that favoured the bimodal Weibull modelling over the single-phase Weibull.

The estimated parameters revealed two interpretable drying phases: fast surface evaporation with a characteristic time constant of $\tau_2 = 4.47 \pm 0.18$ h and the curve shape parameter $\beta_2 = 3.19 \pm 0.15$, accounting for 55.7 ± 2.3% of surface water loss (amplitude parameter A_2), followed by a slower secondary evaporation state with $\tau_1 = 7.14 \pm 0.35$ h and $\beta_1 = 1.12 \pm 0.07$, accounting for 22.0 ± 1.8% of water loss (A_1). The contrasting shape parameters β provide insights into the drying mechanisms: A β_2 of 3.19, far above the pure Fickian diffusion, indicates an accelerated initial drying phase, consisting of rapid surface evaporation following a brief lag period as external water becomes available for removal. In contrast, a β_1 of 1.13 indicates near-Fickian diffusion (near a value of 1) for the slow phase, typical of diffusion-controlled water transport from the gel structure. The final plateau stabilizes at approximately 22% residual bound water. This biphasic behaviour reflects the transition from convection-dominant surface drying to diffusion-limited bulk water removal.

The 95% PI (pink-shaded band), estimated from pooled data across 621 individual measurements from 27 gels, exhibited time-varying bandwidth, being wide during the initial rapid drying phase (0–6 h, ±6–8%) where gel-to-gel variability is the highest, then narrowing substantially during the diffusion-controlled plateau (>10 h, ±2–3%) as all gels converge toward similar equilibrium water content. This heteroscedastic variance pattern reflects the physical nature of the drying process: the early surface-dominated phase is more susceptible to minor differences in gel positioning, local airflow conditions, and surface area variations, while the later diffusion-limited phase is governed by internal transport properties that are more consistent across gels. Importantly, the empirical prediction intervals achieved 93.2% coverage of individual observations. Residual analysis revealed no systematic patterns.

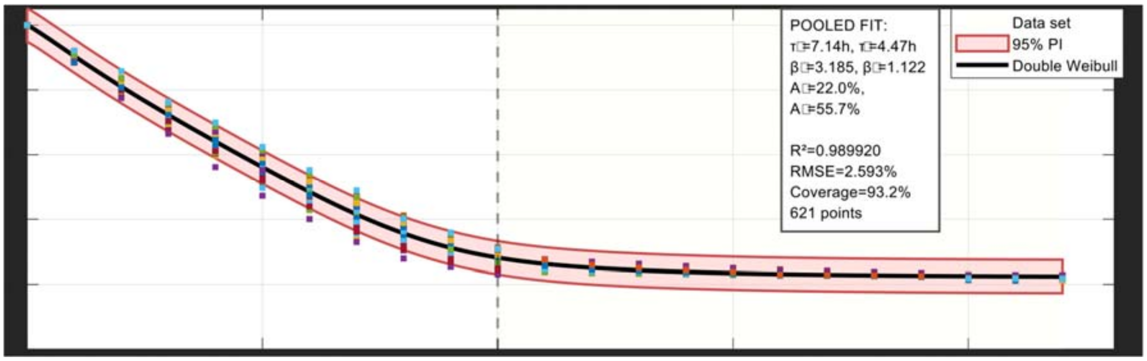

Figure S2. Bimodal Weibull fit for hydrogel drying kinetics. The black line represents the fitted mean drying curve from the bimodal Weibull model, capturing two distinct phases: rapid initial drying and slower diffusion-limited removal. Individual data points represent measurements from 27 gels. The pink-shaded band shows the 95% PI, reflecting the expected range of drying behaviour for newly prepared individual gels and capturing gel-to-gel variability.

Table S8. Estimated parameters of the bimodal Weibull model and corresponding performances metrics. τ_1 and τ_2 represent the characteristic time constants for the fast and the slow evaporation phases, and β_1 and β_2 the dimensionless shape parameters, describing the sigmoidal profile observed experimentally for the fast phase and the exponential near-Fickian profile for the slow phase, explaining the acceleration phase followed by a progressive slowdown of drying, while A_1 and A_2 represent the amplitude of water loss in % for both phases.

Parameter	Value ± SE	Physical Interpretation
**Fast Phase**		Surface evaporation (sigmoidal)
τ_2	4.47 ± 0.18 h	Time constant
β_2	3.19 ± 0.15	Sigmoidal shape (super-Fickian)
A_2	55.7 ± 2.3%	Amplitude (major water loss)
**Slow Phase**		Bulk diffusion (exponential)
τ_1	7.14 ± 0.35 h	Time constant
β_1	1.12 ± 0.07	Near-Fickian diffusion
A_1	22.0 ± 1.8%	Amplitude (bound water)
**Equilibrium**		
$y\infty$	~22%	Final residual water
**Fit Quality**		
R^2	0.990	Coefficient of determination
**RMSE**	2.59%	Root mean square error
**Coverage**	93.2%	Points within 95% PI
n	621	Total measurements (27 gels)

Residual analysis in Figure S3 supported the descriptive adequacy of this current model: residuals of all individual gels were scattered around zero across the fitted range, without a systematic trend or curvature. No overfitting of underfitting was observed across all the gels, suggesting no model misspecification. Uniform vertical spread of residuals over most of the range, with modest widening at the very-high-fitted values, is consistent with the experimental dataset. Results do not reveal persistent gel-specific bias. With the lowest RMSE at the late time points of around 8.0%, observations are compatible with the expected weighing variability and small between gel differences.

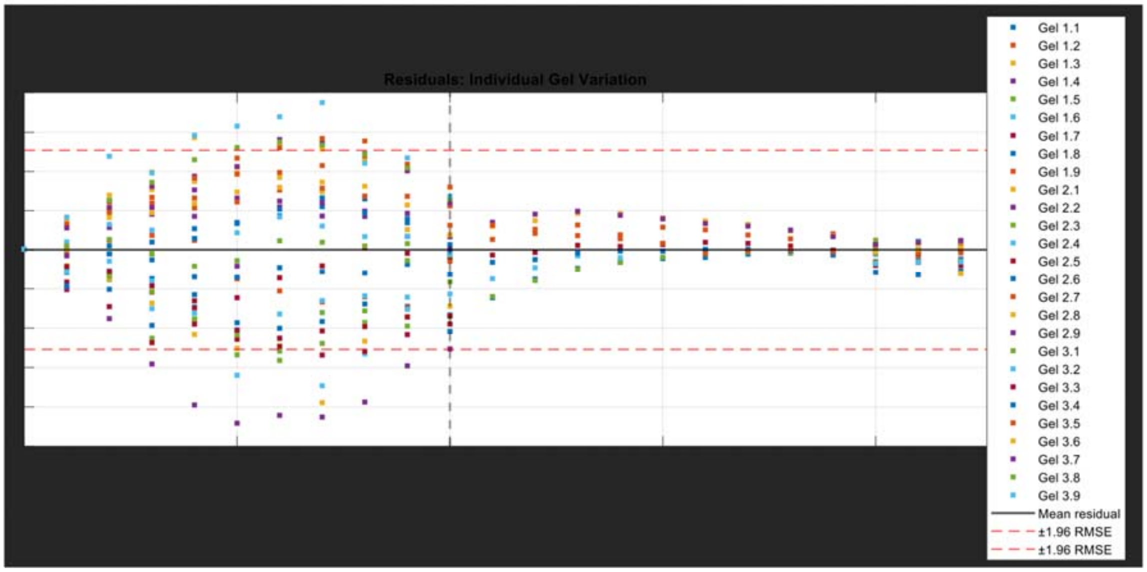

Figure S3. Residuals versus fitted values for the bimodal no-lag Weibull model. Each point represents the residual for one measurement, colour-coded by the gel replicate. Residuals are randomly distributed around the horizontal line at zero, indicating no systematic pattern. The model adequately captures the mean drying curvature, with the vertical spread reflecting experimental variability and minor gel-to-gel differences. Legend: gel x.y (x indicates the batch number; y indicates the replicate number).

Supplementary note S5. Mixed modelling for drying repeatability: covariance structure selection

The most appropriate correlation structure for modelling repeated drying measurements was identified through the evaluation of several candidate structures. Each model was fitted with the same fixed and random effects to ensure comparability, and structures were classified based on Akaike's Information Criterion (AIC) and Bayesian Information Criterion (BIC). These indices balance the model fit, with lower AIC/BIC values indicating a better compromise between explanatory power (goodness-of-fit) of the mixed model and the complexity of the latter. For both indices, differences larger than 4 units are typically regarded as strong evidence favouring the model with the lower criterion.

Table S9. Akaike (AIC) and Bayesian (BIC) information criteria for alternative within-gel covariance structures and comparisons restricted to converged models fitted with identical fixed and random effects. Lower AIC and BIC values indicate superior balance between model fit and parsimony.

Covariance structure of mixed model	AIC	BIC
Homogeneous antedependence	**-653.9**	**-587.1**
Heterogeneous Toeplitz	-593.4	-517.5
Compound symmetry	-561.7	-517.0
Heterogeneous compound symmetry	-532.4	-464.4
AR(1)	-355.6	-339.3

As displayed in Table S9, model performance comparison showed that the homogeneous antedependence structure offered the best description of the within-gel covariance pattern (AIC = -653.9), with improvements of more than 60 units in AIC compared to the heterogeneous Toeplitz (AIC = -593.4). Similarly, large differences were observed relative to compound symmetry (δAIC = 92.3; δBIC = 71.7) and AR(1) (δAIC = 298.3; δBIC = 247.8). These differences largely exceed the threshold of 4 units [8], confirming that the antedependence model provides a better representation of the correlation among the repeated measurements.

Other structures, such as compound symmetry which assumes equal correlations between all observations, is unrealistic for a progressive, time-dependent process. However, compound symmetry can be interesting in the case of simpler modelling data, where the aim is to extract the global tendencies, without overparameterization. AR(1) captures exponential decay in correlation but assumes constant variance across time, which is not in adequation with observed heteroscedasticity in the datasets. Toeplitz structures allowed unequal correlations but were lass parsimonious and penalized by BIC due to the high number of estimated parameters.

Based on the selection of the most adequate model covariance structure, a two-in-one approach was followed for the assessment of gel variability across samples (replicates) and across batches (per day).

Supplementary note S6. Model adequacy evaluation

The quantile–quantile (QQ) plot of conditional residuals in Figure S4 confirms the accuracy of the model assumption. The empirical quantiles closely follow the theoretical normal distribution line, remaining well within the 95% confidence interval. Deviations at the tails are consistent with random noise rather than systematic bias. The accompanying histogram of residuals displays a unimodal distribution centred near zero, confirming once again homoscedasticity and normality assumptions, validating the reliability of the estimated fixed and random effects.

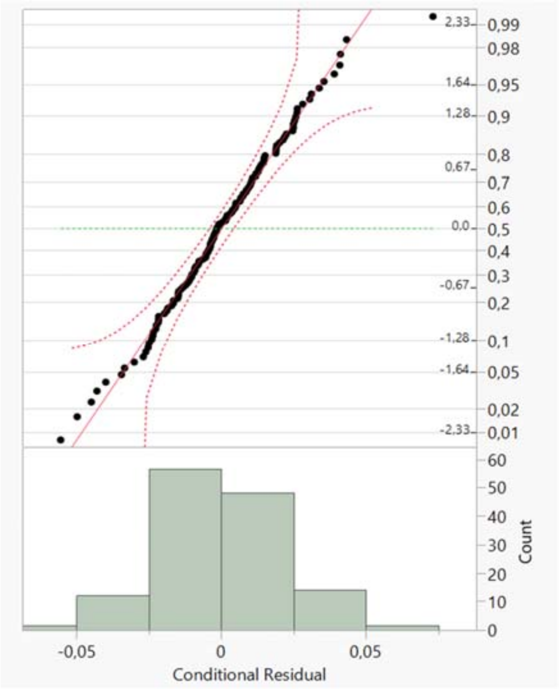

Figure S4. Model adequacy diagnostic for the mixed-effect model describing log-transformed relative water content in hydrogels using the ANTE-EV structure for the inter-day variability evaluation. The QQ plot and histogram of conditional residuals demonstrating normality; data points follow the normal distribution line and remain within the 95% confidence intervals, validating the normality assumption.

Supplementary note S7. SERS imaging data

Data acquired for drop-casting SER-CI testing with different concentrations of diphenhydramine hydrochloride are displayed in Table S9.

Table S10. Data table of mean SERS intensity, standard deviation and relative standard deviation from analysis of dried hydrogels containing different concentrations of diphenhydramine hydrochloride, from 0.1 to 10 mg·mL $^{-1}$. Deposition of AgNPs by drop-casting.

Concentration of diphenhydramine hydrochloride (mg.mL-1)	Mean SERS intensity	Standard deviation	Relative standard deviation (%)
0.1	154.46	57.58	37.34
1	184.57	76.55	41.47
10	264.49	84.78	32.05

References

[1] B. Adnađević, B. Janković, Lj. Kolar-Anić, and D. Minić, "Normalized Weibull distribution function for modelling the kinetics of non-isothermal dehydration of equilibrium swollen poly(acrylic acid) hydrogel," *Chemical Engineering Journal*, vol. 130, no. 1, pp. 11–17, May 2007, doi: 10.1016/j.cej.2006.11.007.

[2] B. Janković, B. Adnađević, and J. Jovanović, "The comparative kinetic study of non-isothermal and isothermal dehydration of swollen poly(acrylic acid) hydrogel using the Weibull probability function," *Chemical Engineering Research and Design*, vol. 89, no. 4, pp. 373–383, Apr. 2011, doi: 10.1016/j.cherd.2010.09.001.

[3] O. Corzo, N. Bracho, A. Pereira, and A. Vásquez, "Weibull distribution for modeling air drying of coroba slices," *LWT - Food Science and Technology*, vol. 41, no. 10, pp. 2023–2028, Dec. 2008, doi: 10.1016/j.lwt.2008.01.002.

[4] X. Zhang, Z. Fan, H. Wu, J. Cong, J. Yang, and B. Wen, "Drying characteristics of green pellets based on the Weibull and Dincer models," *Journal of Safety and Sustainability*, vol. 2, no. 2, pp. 104–112, Jun. 2025, doi: 10.1016/j.jsasus.2025.05.001.

[5] M. Torki-Harchegani, D. Ghanbarian, and M. Sadeghi, "Estimation of whole lemon mass transfer parameters during hot air drying using different modelling methods," *Heat Mass Transfer*, vol. 51, no. 8, pp. 1121–1129, Aug. 2015, doi: 10.1007/s00231-014-1483-1.

[6] M. M. M. Hasan, R. Ara, L. C. Shaha, A. Sarkar, and M. Alam, "Modeling the drying behavior and mass transfer phenomena in osmotically dehydrated tomatoes," *Food Chemistry Advances*, vol. 8, p. 101093, Sep. 2025, doi: 10.1016/j.focha.2025.101093.

[7] A. Sánchez-Ferrer, M. Engelhardt, and K. Richter, "Anisotropic wood–water interactions determined by gravimetric vapor sorption experiments," *Cellulose*, vol. 30, no. 6, pp. 3869–3885, Apr. 2023, doi: 10.1007/s10570-023-05093-z.

[8] K. P. Burnham and D. R. Anderson, "Multimodel Inference: Understanding AIC and BIC in Model Selection," *Sociological Methods & Research*, vol. 33, no. 2, pp. 261–304, Nov. 2004, doi: 10.1177/0049124104268644.
